# An efficient Bayesian observer model of attractive and repulsive temporal context effects when perceiving multistable dot lattices

**DOI:** 10.1167/jov.24.4.18

**Published:** 2024-04-18

**Authors:** Eline Van Geert, Tina Ivančir, Johan Wagemans

**Affiliations:** 1 Laboratory of Experimental Psychology, Department of Brain and Cognition, KU Leuven, Belgium

**Keywords:** efficient coding, hysteresis, adaptation, individual differences, perceptual organization, multistable perception, serial dependencies, context effects, attraction, repulsion

## Abstract

In multistable dot lattices, the orientation we perceive is attracted toward the orientation we perceived in the immediately preceding stimulus and repelled from the orientation for which most evidence was present previously (Van Geert, Moors, Haaf, & Wagemans, 2022). Theoretically-inspired models have been proposed to explain the co-occurrence of attractive and repulsive context effects in multistable dot lattice tasks, but these models artificially induced an influence of the previous trial on the current one without detailing the process underlying such an influence (Gepshtein & Kubovy, 2005; Schwiedrzik et al., 2014). We conducted a simulation study to test whether the observed attractive and repulsive context effects could be explained with an efficient Bayesian observer model (Wei & Stocker, 2015). This model assumes variable encoding precision of orientations in line with their frequency of occurrence (i.e., efficient encoding) and takes the dissimilarity between stimulus space and sensory space into account. An efficient Bayesian observer model including both a stimulus and a perceptual level was needed to explain the co-occurrence of both attractive and repulsive temporal context effects. Furthermore, this model could reproduce the empirically observed strong positive correlation between individuals’ attractive and repulsive effects (Van Geert et al., 2022), by assuming a positive correlation between temporal integration constants at the stimulus and the perceptual level. To conclude, the study brings evidence that efficient encoding and likelihood repulsion on the stimulus level can explain the repulsive context effect, whereas perceptual prior attraction can explain the attractive temporal context effect when perceiving multistable dot lattices.

## Introduction

What we perceive is not only influenced by the current stimulus we have in front of our eyes, but also by the recent stimulus and perceptual history. Many recent studies have confirmed the existence of both attractive and repulsive effects of immediate temporal context on perception (Bosch, Fritsche, Ehinger, & de Lange, 2020; Fritsche, Spaak, & de Lange, 2020; Pascucci et al., 2019; Sadil, Cowell, & Huber, 2024; Snyder, Schwiedrzik, Vitela, & Melloni, 2015; Van Geert et al., 2022). Also in the perception of multistable dot lattices, both attractive and repulsive context effects are at play (Gepshtein & Kubovy, 2005; Schwiedrzik et al., 2014; Van Geert et al., 2022). On the one hand, the perceived orientation in these lattices is attracted toward the orientation perceived in the immediately preceding lattice (i.e., hysteresis, attractive effect of previous percept). On the other hand, the perceived orientation is repelled from the orientation for which most evidence was present in the previous lattice (i.e., adaptation, repulsive effect of previous stimulus evidence, Gepshtein & Kubovy, 2005; Schwiedrzik et al., 2014; Van Geert et al., 2022).

Several theoretically inspired models have been proposed to explain the co-occurrence of these context effects when perceiving multistable stimuli (Gepshtein & Kubovy, 2005; Schwiedrzik et al., 2014), but these models either artificially induce a direct influence of the previous stimulus evidence on the likelihood distribution for the current stimulus (Schwiedrzik et al., 2014) or induce a randomly determined shift in prior bias from the previous to the current percept (Gepshtein & Kubovy, 2005). From these models, it is not clear why such a direct influence would occur, or which underlying process would determine a random shift in bias. In this simulation study, we investigate whether an efficient Bayesian observer model based on Wei and Stocker (2015) can explain the co-occurrence of both attractive and repulsive temporal context effects in multistable dot lattice perception. Earlier variants of the efficient Bayesian observer model have successfully been used to model effects in different tasks involving non-ambiguous stimuli (Fritsche et al., 2020; Langlois, Jacoby, Suchow, & Griffiths, 2021; Wei & Stocker, 2015). In this study, we assess the viability of explaining temporal context effects on multistable dot lattice perception using an efficient Bayesian observer model. As part of this investigation, we test whether the model can not only successfully account for the average temporal context effects observed in Van Geert et al. (2022), but also for the observed range and strong positive correlation of interindividual variation in both effects.

### Attractive and repulsive temporal context effects: Separate but related mechanisms?

Whereas repulsive temporal context effects are often seen as resulting from the previous stimulus evidence, attractive temporal context effects are seen as resulting from the previous percept, response, and/or decision (Bosch et al., 2020; Sadil et al., 2024; Schwiedrzik et al., 2014; Van Geert et al., 2022). This is often related to repulsion being a more “low-level” phenomenon, showing larger spatial and featural specificity than the “higher-level” attraction (Fritsche et al., 2020; Gepshtein & Kubovy, 2005; Schwiedrzik et al., 2014). Schwiedrzik et al. (2014) also found both effects to map into distinct cortical networks. Although many researchers thus state that attractive and repulsive effects result from separate processes (Brascamp et al. 2008; Fritsche et al., 2020; Pascucci et al., 2019; Schwiedrzik et al., 2014), others argue that both effects share a common underlying mechanism (Gepshtein & Kubovy, 2005; Mattar, Kahn, Thompson-Schill, & Aguirre, 2016; Maus, Chaney, Liberman, & Whitney, 2013). Because Van Geert et al. (2022) found a strong positive correlation between the size of individuals’ hysteresis (i.e., attractive effect of previous percept) and adaptation effects (i.e., repulsive effect of previous stimulus evidence), there needs to be at least some common factor influencing both effects. On the other hand, the results of Van Geert et al. (2022) did not bring evidence for a completely unified process underlying both effects, as the correlation estimate (already corrected for attenuation) was only *r* = 0.68 (95% highest density continuous interval [HDCI], 0.54–0.79; cf. Figure 10a). We therefore hypothesize that both effects stem from separate but related mechanisms, and in this simulation study an efficient Bayesian observer model is put forward to model the processes underlying both context effects in a theoretically coherent way.

**Figure 1.**
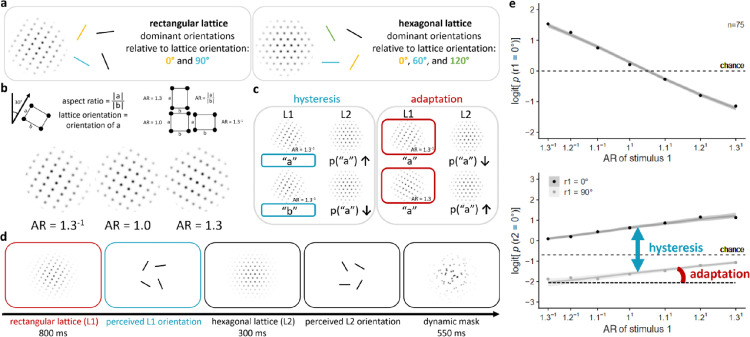
(a) Dominantly perceived orientations in multistable rectangular and hexagonal dot lattices. (b) Explanation regarding the AR of a multistable rectangular dot lattice. In rectangular dot lattices, four different orientations can be perceived, of which two are more prevalent (as the dots are closer together along these orientations). The relative dominance of the a orientation relative to the b orientation is expressed in the AR of the dot lattice (AR = |a| / |b|). (c) Illustration of attractive and repulsive context effects in dot lattices. Left side: attraction effect (hysteresis). When the first lattice (L1) is *perceived* as orientation a (indicated by “a”), the probability that the second lattice (L2) will be perceived as orientation a is higher than when L1 was interpreted as orientation b (indicated by “b”). Right side: repulsion effect (adaptation). When strong support for orientation a is present in L1, the probability that L2 will be perceived as orientation a is lower than when L1 had less support for orientation a. (d) Illustration of trial structure. For reasons of visibility, the shown trial components in this figure have black dots on a white background. The actual experiment had white dots on a grey background. (e) Mean empirical logit probability of perceiving the relative 0° orientation in the first and the second lattice dependent on the AR. The probability of responding 0° to the first lattice decreases with the AR (|a|/|b|). The value of the AR increases with increasing distance in the 0°-orientation, leading to more 90° responses. The probability of responding 0° to the second lattice increases with the AR (|a|/|b|; i.e., adaptation effect), and increases when the first stimulus was perceived as 0° rather than 90° (i.e., hysteresis effect). Reprinted and adapted from Van Geert et al. (2022).

**Figure 2.**
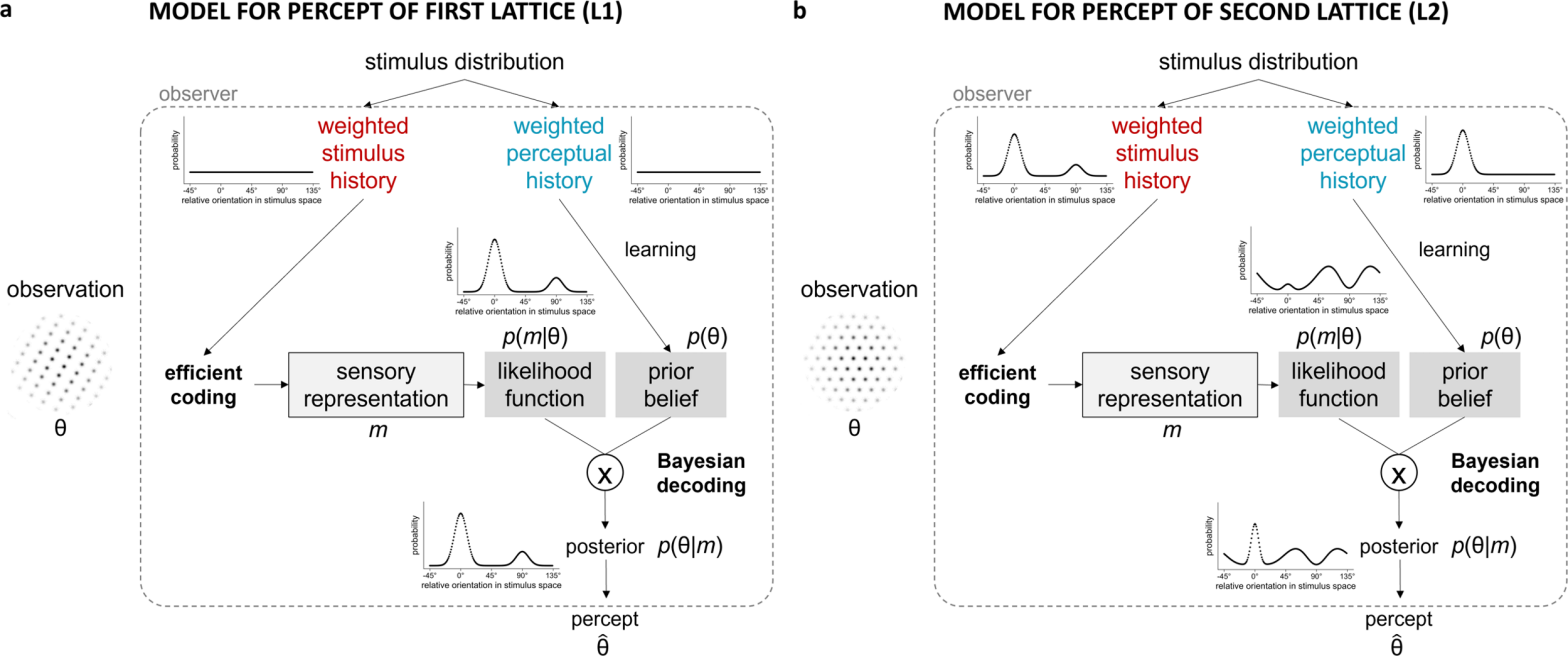
Schematic representation of the efficient Bayesian observer model for the percept of (a) the first lattice and (b) the second lattice, with a uniform prior for the first lattice and the following parameter values: *c*_stim_ = 5, κ_stimL1_ = 20, κ_sensL1_ = 20, κ_stimL2_ = 20, κ_sensL2_ = 18, κ_percL1_ = 10, *w*_stimL1_ = 0.60, and *w*_percL1_ = 0.50.

**Figure 3.**
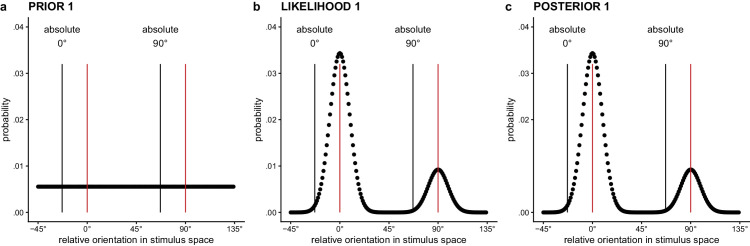
(a) Uniformly distributed prior for the first lattice. (b) Likelihood distribution defined in the stimulus space, for a first lattice with an absolute lattice orientation of 23° and AR = 1.3^−1^, which favors the relative 0° orientation. (c) Posterior distribution for the first lattice. Based on the difference in height of the peaks for the relative 0° and 90° orientation, i.e., p(0°) and p(90°), the probability of a 0° or 90° response can be determined. *Note.* The red vertical lines in the graph are placed at the two dominant relative 0° and 90° orientations in the lattice. The black vertical lines label the absolute 0° and 90° orientations.

**Figure 4.**
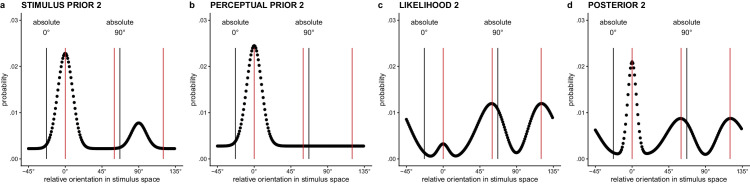
(a) Stimulus prior for the second lattice given a first lattice with AR = 1.3^−1^, which favors the relative 0° orientation. (b) Perceptual prior for the second lattice, given the relative 0° orientation was perceived in the first lattice. (c) Likelihood distribution defined in the stimulus space for the second lattice. This distribution is influenced by the stimulus prior for the second lattice (and hence the AR of the first lattice) via the stimulus-to-sensory mapping. (d) Posterior distribution for the second lattice, combining perceptual prior and likelihood for the second lattice. Based on the difference in height of the peaks for the relative 0°, 60°, and 120° orientation, the probability of a 0°, 60°, or 120° response can be determined. *Note.* The red vertical lines in the graph are placed at the three dominant relative 0°, 60°, and 120° orientations in the lattice. The black vertical lines label the absolute 0° and 90° orientations.

**Figure 5.**
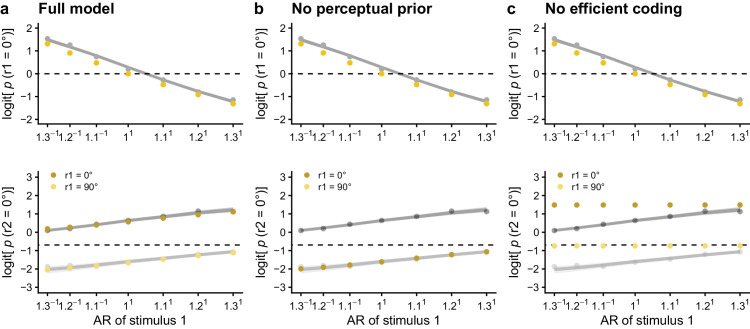
Visualization of the logit probability to perceive the relative 0° orientation in the first lattice and the second lattice, based on (a) an efficient Bayesian observer model with a flat prior distribution for the first lattice and the following parameters: *c*_stim_ = 5, κ_stimL1_ = 20, κ_sensL1_ = 20, κ_stimL2_ = 20, κ_sensL2_ = 18, κ_percL1_ = 10, *w*_stimL1_ = 0.60, and *w*_percL1_ = 0.50, (b) the same model as in (a), but using a stimulus prior rather than a perceptual prior for the second lattice and with *w*_stimL1_ = 0.60, and *w*_percL1_ = 0, and (c) the same model as in (a), but without efficient encoding. The yellow dots indicate the expected probabilities based on the model. In (b), the dark and light yellow dots lay on top of each other. The behavioral results and the estimated effects based on the behavioral results of Van Geert et al. (2022), averaged across participants, are indicated in dark grey for r1 = 0° and light grey for r1 = 90°.

**Figure 6.**
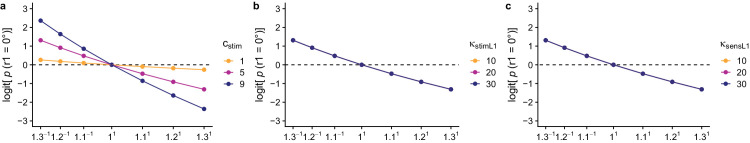
Effects of parameter variations on the logit probability of perceiving the relative 0° orientation in the first lattice, for an efficient Bayesian observer model with a uniform prior for the first lattice and baseline parameter values: *c*_stim_ = 5, κ_stimL1_ = 20, and κ_sensL1_ = 20. Under these settings, κ_stimL1_ and κ_sensL1_ do not influence the size of the direct proximity effect (i.e., the effect of the AR on the percept of the first lattice).

**Figure 7.**
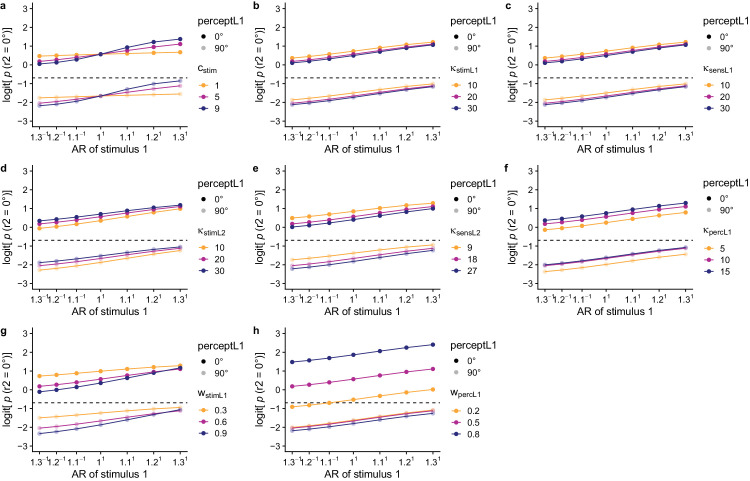
Effects of parameter variations on the logit probability of perceiving the relative 0° orientation in the second lattice, for an efficient Bayesian observer model with a uniform prior for the first lattice and baseline parameter values: *c*_stim_ = 5, κ_stimL1_ = 20, κ_sensL1_ = 20, κ_stimL2_ = 20, κ_sensL2_ = 18, κ_percL1_ = 10, *w*_stimL1_ = 0.60, and *w*_percL1_ = 0.50.

**Figure 8.**
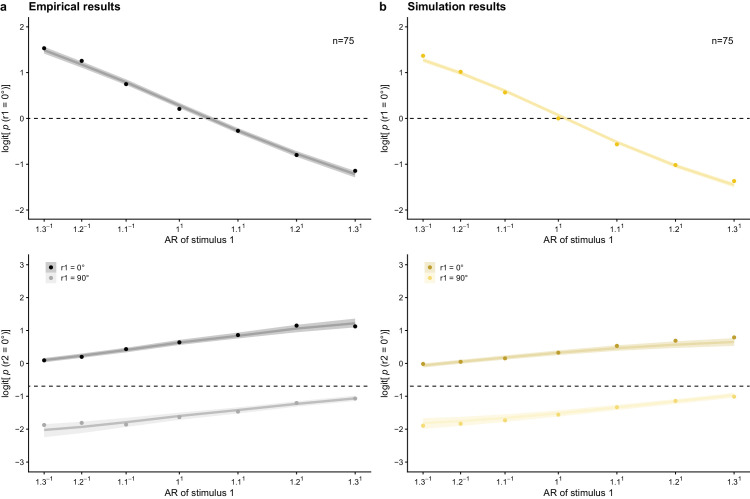
(a) Mean empirical logit probability of perceiving the relative 0° orientation in the first and the second lattice dependent on the AR. The probability of responding 0° to the first lattice decreases with the AR (|a|/|b|). The value of the AR increases with increasing distance in the 0°-orientation, leading to more 90° responses. The probability of responding 0° to the second lattice increases with the AR (|a|/|b|; i.e., adaptation effect), and increases when the first stimulus was perceived as 0° rather than 90° (i.e., hysteresis effect). (b) Mean simulated logit probability of perceiving the relative 0° orientation in the first and the second lattice dependent on the AR. *Note.* Dots indicate mean values. In addition, mean posterior predictions and their 95% highest density continuous intervals are shown.

**Figure 9.**
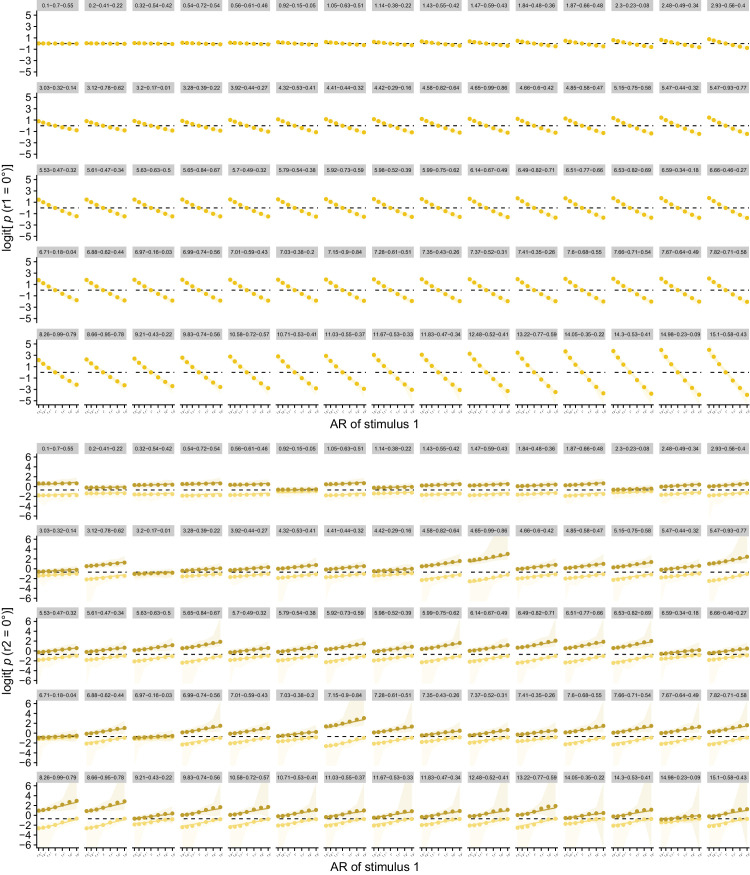
(a) Mean simulated individual responses to the first stimulus dependent on the AR (logit). The probability of responding 0° to the first stimulus decreases with the AR (|a|/|b|). The value of the AR increases with increasing distance in the 0°-orientation, leading to more 90° responses. Dots indicate observed values. In addition, mean posterior predictions and their 95% highest density continuous intervals are shown. (b) Mean simulated individual responses to the second stimulus dependent on the AR (logit). The probability of responding 0° to the second stimulus increases with the AR (|a|/|b|; i.e., adaptation effect), and increases when the first stimulus was perceived as 0° rather than 90° (i.e., hysteresis effect). Dots indicate observed values. In addition, mean posterior predictions and their 95% highest density continuous intervals are shown. *Note.* Labels indicate parameter values for *c*_stim_, *w*_stimL1_, and *w*_percL1_ per simulated participant, respectively.

**Figure 10.**
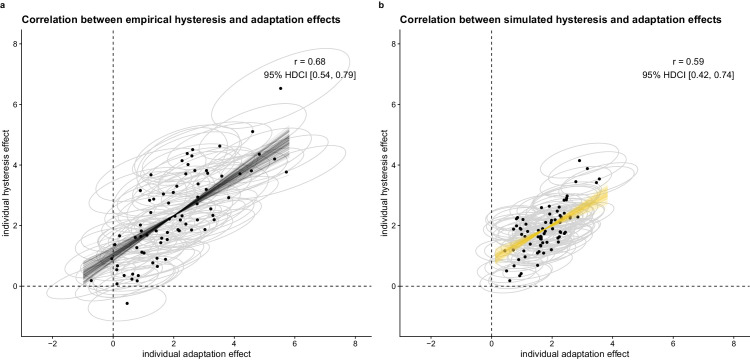
Correlation between individual slopes for the effect of the AR and perceived L1 orientation on perceiving the 0° orientation in L2, for (a) the empirical data collected in Van Geert et al. (2022) and (b) the simulated data. Mean and 80% highest density continuous intervals (HDCI) are shown. The dashed lines indicate a slope of zero. The black and yellow lines give examples of plausible correlation estimates.

### Attractive and repulsive temporal context effects in multistable dot lattice perception

Gepshtein and Kubovy (2005) proposed a paradigm to distinguish between attractive and repulsive context effects on perception. They investigated the influence of (a) the perceived organization of the preceding stimulus (i.e., which organization was reported) and (b) the stimulus support for a certain organization in the preceding stimulus (dependent on the stimulus aspect ratio [AR]) on the perception of a second, current stimulus, using multistable dot lattices as stimuli.

Multistable dot lattices are aligned dot arrays in which multiple orientations can be perceived. In rectangular dot lattices, four different orientations can be perceived (cf. left part of Figure 1a), two of which are more prevalent. In hexagonal dot lattices (cf. right part of Figure 1a), three equally plausible orientations are most prominent. According to the Gestalt law of proximity (Kubovy, Holcombe, & Wagemans, 1998), the closer the dots are together along a particular orientation, the more likely they will be grouped together, and consequently, the more likely that orientation will be perceived. Relative grouping strength has been shown to decrease exponentially in accordance with the relative inter-dot distance (Kubovy et al., 1998). For two orientations a and b, the AR of a dot lattice (AR = |a|/|b|) expresses the a orientation’s relative dominance over the b orientation (cf. Figure 1b). For a lattice with AR = 1, the inter-dot distance in the a and b orientations is equal. For a lattice with an AR of less than 1, the inter-dot distance is smaller in the a than in the b orientation. For a lattice with an AR of more than 1, the inter-dot distance is smaller in the b than in the a orientation. In both rectangular and hexagonal dot lattices, we define the axis orientation of the dot lattice as a whole by the a orientation, which we will refer to as the 0° orientation. In the rectangular dot lattices, we will refer to the b orientation as the 90° orientation.

The multistable dot lattice paradigm introduced by Gepshtein and Kubovy (2005) to concurrently assess attractive and repulsive immediate temporal context effects on perception was later adapted by Schwiedrzik et al. (2014, cf. Figure 1d). They used rectangular dot lattices with randomly varying absolute lattice orientation as context stimuli (presented for 800 ms) and more ambiguous hexagonal dot lattices with the same random absolute lattice orientation as test stimuli (presented for 300 ms). Participants indicated which orientation they perceived in each lattice using a four-alternative forced-choice task (always including the most dominant orientations). To manipulate the stimulus support for the 0° orientation, the AR of the rectangular dot lattice was varied. They then assessed how (a) perceived orientation and (b) the AR in the first, rectangular lattice affected perceived orientation in the second, hexagonal lattice. An attractive (i.e., hysteresis) effect of the previous percept was present, as well as a repulsive (i.e., adaptation) effect of the previous stimulus evidence: the probability of perceiving a particular orientation in the second lattice increased when the same orientation was perceived in the first lattice, and the stronger the evidence for a specific orientation in the first lattice, the lower the probability to perceive that orientation in the second lattice (cf. Figure 1c). These results by Gepshtein and Kubovy (2005) and Schwiedrzik et al. (2014) were replicated and extended by Van Geert et al. (2022, cf. Figure 1e). Whereas Gepshtein and Kubovy (2005) and Schwiedrzik et al. (2014) mainly explored the existence of these effects on the group level, Van Geert et al. (2022) tested whether individual differences existed in the size of these effects, and whether every individual participant showed both effects in the expected direction. The results confirmed the presence of large, consistent differences in the size of attractive and repulsive context effects across individuals, and these differences stayed stable across 1 to 2 weeks time. Furthermore, almost every participant showed both effects in the expected direction, although not every single participant did. As indicated earlier in this article, the results of Van Geert et al. (2022) provided evidence for at least some common factor underlying both effects, as individual differences in attractive and repulsive context effects were highly positively correlated (cf. Figure 10a). Also, hysteresis (i.e., the attractive effect of the previous percept) showed to be a partially percept-related and a partially decision-related effect, nuancing earlier debates on the origin of this effect (Bosch et al., 2020; Cicchini, Mikellidou, & Burr, 2017; Fritsche, Mostert, & de Lange, 2017; Manassi, Liberman, Kosovicheva, Zhang, & Whitney, 2018; Pascucci et al., 2019; Schwiedrzik et al., 2018).

Now that the existence of both temporal context effects on multistable dot lattice perception has been firmly established, including consistent variation in the size of the effects across individuals, one way forward is to further our understanding of the processes underlying these effects by developing models and verifying whether they can reproduce and explain the range of variability in effect size and direction across individuals. Importantly, a good process model should not only be able to predict a mean response, but also plausible variation in the effect size and direction (Van Geert et al., 2022).

### Models of temporal context effects on multistable dot lattice perception

Two earlier models of the multistable dot lattice paradigm for hysteresis and adaptation exist (Gepshtein & Kubovy, 2005; Schwiedrzik et al., 2014). In the model of Gepshtein and Kubovy (2005), combinations of attraction strengths (related to inter-dot distances in the first, rectangular lattice and sensitivity to the inter-dot distance) and a randomly determined persistent intrinsic bias (i.e., a higher probability to perceive some orientation more than others, which stays similar but not identical from first to second lattice) determine the perceived orientation in the second, hexagonal lattice. When the intrinsic bias exceeds the stimulus support (i.e., attraction strength), the multistable lattice is perceived inconsistently with the stimulus support. In this model, the direction of the intrinsic bias is determined randomly, and it is not clear where the bias comes from or which process determines the direction of the bias.

Schwiedrzik et al. (2014) developed a Bayesian model to account for the co-occurrence of hysteresis and adaptation in multistable dot lattice perception. They model both effects independently, with the perceived orientation in the first lattice directly impacting the prior distribution of possible perceived orientations for the second lattice, and the reduction in stimulus support due to neuronal adaptation to the AR of the first lattice directly impacting the likelihood distribution for the second lattice. In this model, the process behind the direct change in the likelihood is not included and its size is determined arbitrarily.

Based on the model by Wei and Stocker (2015), we propose an alternative Bayesian model that can explain the co-occurrence of hysteresis and adaptation as separate but related processes influencing multistable dot lattice perception. A similar model has been applied by Fritsche et al. (2020) to explain attractive and repulsive stimulus history effects in orientation perception. They did look at the influence of previous stimuli and went more than one stimulus back, but did not distinguish between the effects of previous percepts and previous stimulus evidence, and only used non-ambiguous Gabor stimuli.

### Efficient Bayesian observer models of perception

A key assumption of an efficient Bayesian observer model is that available coding resources are limited, and that those feature values that occur more frequently will be more accurately encoded or represented (i.e., the principle of efficient coding, Wei, & Stocker, 2015). In case of orientation perception, this variable encoding precision will thus lead to frequent orientations being encoded more accurately than less frequent orientations. A second key feature of the model is that it takes the dissimilarity between stimulus space and sensory space into account (as in psychophysics). This leads to differential predictions of adding external stimulus noise, that is, noise related to uncertainty in a specific stimulus feature (e.g., variance in orientation) or internal sensory noise, that is, noise related to uncertainty in encoding and processing (e.g., due to presentation duration, stimulus size, or luminance contrast). Whereas external stimulus noise will only widen the likelihood distribution and increase the overall influence of the prior (leading to stronger prior attraction, “Bayesian” percepts), internal sensory noise symmetric around the stimulus value in sensory space will make the likelihood distribution asymmetric in stimulus space and hence create the possibility for biases away from the peak of the prior distribution (i.e., likelihood repulsion, “anti-Bayesian” percepts, Wei, & Stocker, 2015). The relative amount of stimulus versus sensory noise will determine which effect will show behaviorally (i.e., attraction or repulsion).

More concretely, frequency of occurrence will jointly influence prior and likelihood in the model. It influences the prior distribution directly: more frequently occurring orientations are also expected to occur more often. However, the frequency of occurrence also influences the mapping between stimulus and sensory space: it influences the accuracy with which different orientations will be encoded, and consequently also the width and form with which different orientations will be represented in the likelihood. In other words, the frequency of occurrence will determine the prior distribution as well as how the currently encountered stimulus will be encoded. The stimulus-to-sensory mapping is given by the cumulative density function of the encoding accuracy distribution, and the sensory-to-stimulus mapping is given by the inverse of that cumulative density function.

In the original model, frequency of occurrence was seen on the long term. For example, in most daily environments, cardinal orientations are more prevalent than oblique ones (Coppola, Purves, McCoy, & Purves, 1998; Girshick, Landy, & Simoncelli, 2011). In later versions of the model, however, it has been shown that frequency of occurrence can also be defined on the short term, e.g., with the frequency of occurrence changing during the experiment (Fritsche et al., 2020; Ni & Stocker, 2023; Noel, Zhang, Stocker, & Angelaki, 2021). For example, Ni and Stocker (2023) demonstrated that robust averaging (i.e., the non-uniform weighting of items in a stimulus ensemble) emerged naturally from an optimal integration process when sensory encoding (and thus the stimulus-to-sensory mapping) was efficiently adapted to the ensemble statistics in the experiment. Their model accurately predicted the dependence of subjects’ decision accuracy and non-uniform weighting profile on the specific stimulus distribution in the experiments. Furthermore, Noel et al. (2021) showed that neurotypical participants adapted their sensory encoding to the changing stimulus statistics when they were exposed to an artificial uniform orientation distribution coupled with performance feedback in a visual orientation estimation task. Changes in the frequency of occurrence on the short term may thus be used to model short-term temporal context effects on perception.

Fritsche et al. (2020) modeled attractive and repulsive biases of stimulus history using an efficient Bayesian observer model. In this model, they did not disentangle effects of previous stimuli and previous percepts, but treated all effects as related to the previous stimulus evidence. In the empirical study they conducted, they found evidence for short-term attraction and long-term repulsion. When fitting different models to the empirically collected data, a model with distinct transition distributions and different integration time constants for prior and likelihood performed better than a model that used the same parameters for prior and likelihood. In the prior distribution, only the most recently presented stimuli mattered, and updating was fast. In the likelihood distribution, information was integrated over longer timescales, and updates happened more slowly.

In a recent extension of the original model proposed by Wei and Stocker (2015), Mao and Stocker (2024) described perception as a holistic inference process, where the percept of a stimulus is jointly represented at different levels of a representational hierarchy. To adequately model the (variation in) behavioral data of an earlier study (Tomassini, Morgan, & Solomon, 2010), it was necessary to take the higher-level representation into account (i.e., categorization of orientation in this case).

### An efficient Bayesian observer model for temporal context effects on multistable dot lattice perception

In this study, we develop an efficient Bayesian observer model for the multistable dot lattices paradigm used by Gepshtein and Kubovy (2005), Schwiedrzik et al. (2014), and Van Geert et al. (2022) to assess hysteresis and adaptation effects (cf. Figure 2 for a schematic overview of the proposed model). In addition, we investigate how several versions of the model compare to the empirical results obtained by Van Geert et al. (2022). More specifically, we test whether model implementations can explain co-occurring attractive and repulsive context effects, as well as a range of plausible variation in effect size and direction across “individuals” (in this case across simulations with different parameter values). Furthermore, we test whether the model can reproduce a positive correlation between the size of both effects, as was empirically observed in Van Geert et al. (2022).

Different from the original Wei and Stocker (2015) model, we will not only implement a long-term orientation prior (at least in some variants of the model), but also take the short-term context into account: the prior and stimulus-to-sensory mapping for the second lattice will be updated based on the stimulus evidence present for and the percept of the first lattice. In line with the earlier efficient Bayesian observer models that take short-term context into account, we assume an influence of the *stimulus history* (i.e., the frequency of occurrence of different stimulus orientations) on the stimulus-to-sensory mapping and consequently the likelihood distribution. In contrast to earlier efficient Bayesian observer models, however, we will argue that it is the *perceptual history* (i.e., the frequency of occurrence of different perceived orientations) rather than the stimulus history that determines the prior distribution. Different from the implementation by Fritsche et al. (2020), the model will distinguish attractive influences of the previous percept and repulsive influences of the previous stimulus evidence. Given that a mask was present in the dot lattice paradigm to avoid longer-term context effects, we only take the previous lattice into account and do not model longer-term context influences (different from what was the case in Fritsche et al., 2020). Furthermore, the dot lattice paradigm concerns multistable stimuli resulting in multi-peaked likelihood distributions, whereas previous implementations of the efficient Bayesian observer model focused on non-ambiguous stimuli (e.g., Fritsche et al., 2020; Wei & Stocker, 2015). In sum, our model builds on earlier models, but makes at least three innovative contributions.

## Methods

### Efficient Bayesian observer model

In this study, we model the perception of two consecutive dot lattices within one trial of the paradigm. All model simulations were performed in R (Version 4.0.4, R Core Team, 2021).^1^ All code related to this paper is openly available on the Open Science Framework: https://doi.org/10.17605/OSF.IO/48ESD.

The first lattice is a rectangular dot lattice with varying AR across trials, the second is a hexagonal dot lattice with three equally dominant orientations. First, we develop the model for the percept of the first lattice. Then, we update the prior and stimulus-to-sensory mapping to predict hysteresis and adaptation effects in the perception of the second lattice. Different from earlier efficient Bayesian observer models, which assume the actual frequency of occurrence of different stimulus values (i.e., the stimulus history) to influence both the prior and the likelihood in the model, we assume the stimulus history to influence the stimulus-to-sensory mapping and the likelihood, but the perceptual history (i.e., the history of how earlier presented stimuli were actually perceived) to influence the prior distribution (i.e., the belief of the observer). When the history contains only non-ambiguous stimuli, stimulus history and perceptual history will approximately align, and ignoring this distinction remains without consequences. We will, for example, assume stimulus history and perceptual history to be similar when we determine the percept of the first lattice in each trial. However, when the history contains multistable stimuli, for example, when determining the percept of the second lattice, this distinction between stimulus history and perceptual history becomes evident and consequential. As will be shown later in this article, a version of the model that used the stimulus history to influence both prior and likelihood could explain the occurrence of adaptation, but not the occurrence of the hysteresis effect. In contrast, the current version of the model, which assumes the stimulus history to influence the likelihood and the perceptual history to influence the prior, is able to predict both co-occurring temporal context effects. The adaptation effect will be due to efficient encoding and likelihood repulsion on the stimulus level, the hysteresis effect will be due to prior attraction on the perceptual level. Therefore, we will describe this model as *hierarchical*. The size of the adaptation effect will depend on the relative amount of stimulus noise and sensory noise present, but the size of both context effects will depend mostly on the weights given to the stimulus evidence and percept in the previous trial compared to the long-term context.

#### Perception of the first lattice

We first model how an observer comes to perceive either the relative 0° or the relative 90° orientation in the first lattice. Over trials, both the AR and the absolute orientation of the lattice are varied. The distribution of absolute lattice orientations in the experiment is uniform, but the long-term natural stimulus distribution of orientations has peaks at both cardinal orientations. As in earlier Bayesian encoding models, we assume encoding resources to be allocated according to the stimulus distribution (uniform in the experiment or with peaks at the cardinals in the long term) so that stimulus values that occur more frequently are more accurately represented. Two types of noise are assumed: external stimulus noise and internal sensory noise. The external stimulus noise (symmetric in stimulus space) is assumed to follow a von Mises (i.e., circular normal) distribution on the 180° (i.e., half-circular) orientation space with its mean at the actual stimulus orientation value in question and its precision being equal to κ_stimL1_. The internal sensory noise (symmetric in sensory space) is expected to follow a von Mises (i.e., circular normal) distribution on the 180° (i.e., half-circular) orientation space with its mean at the sensory measurement for the actual stimulus orientation in question (based on the stimulus-to-sensory mapping, derived from the cumulative density function for the prior distribution) and its precision being equal to κ_sensL1_. The described stimulus and sensory noise are jointly reflected in the noise of the observer’s representation of the stimulus orientation. The observer’s representation of the stimulus orientation (subject to the stimulus noise and sensory noise described earlier in this article) is expected to be bimodal, with peaks at the relative 0° and the relative 90° orientation.^2^ The relative height of the peaks at the relative 0° and the relative 90° orientation will depend on the AR of the stimulus and the observer’s sensitivity for AR. This bimodal distribution represents the likelihood, and is combined with the prior distribution (either uniform in stimulus space or with peaks at the cardinal orientations) to compute the posterior distribution for the first lattice. From the posterior distribution, either a relative 0° or a relative 90° percept can be sampled with the probabilities depending on the relative probability of perceiving one versus the other orientation.

#### Prior distribution for the first lattice

In the prior distribution for the first lattice, the long-term perceptual distribution for orientation is represented. For this first lattice, we assume the long-term perceptual distribution for orientation to be equal to the long-term stimulus distribution for orientation. We try two variants of the prior. In a first variant, we use the same natural stimulus distribution as in Wei and Stocker (2015):

(1) $$p\left(\theta \right)=c_{0}\left(2-|sin\left(2\theta \right)|\right),$$

where *c*_0_ is a normalization constant and θ ∈ [0, π) (cf. Supplementary Figure 1a). This natural stimulus distribution reflects the fact that horizontal and vertical orientations are more common in the natural environment than oblique orientations. On the other hand, within the dot lattice paradigm the long-term stimulus distribution is uniform: every absolute lattice orientation occurs equally frequently. Therefore, we implemented a second variant of the model, with a uniform prior distribution for the first lattice:

(2) $$p\left(\theta \right)=\frac{1}{\pi }$$

(cf. Figure 3a). The distribution used in this prior for the first lattice will also affect the stimulus-to-sensory mapping that is used in the calculation of the likelihood distribution for the first lattice.

#### Likelihood function for the first lattice

Given that the first dot lattice is rectangular, it has two dominant orientations, of which the relative dominance is dependent on the AR of the lattice and the observer’s sensitivity to AR. Here we assume the observer’s representation of the first lattice to be a weighted combination of the expected sensory measurement for the 0° orientation and the expected sensory measurement for the 90° orientation. In case AR = 1, we expect the stimulus support to be equal for both orientations, which will be represented by an equal weight for both likelihood functions. In case AR ≠ 1, one of the two sensory measurements will have a stronger representation in the combined likelihood than the other. To arrive at a double-peaked likelihood (cf. Figure 3b), we combine the likelihoods for both sensory measurements (i.e., the expected sensory measurements for the relative 0° and the relative 90° orientation) in a weighted fashion:

(3) $$\begin{array}{c} \;p\left(m_{L1}|\theta \right)\propto p\left(m_{0}|\theta \right)\cdot \frac{1}{1+w_{AR}}+p\left(m_{90}|\theta \right)\cdot \frac{w_{AR}}{1+w_{AR}},\;\;\;\;\; \end{array}$$

with *p*(*m*_0_|θ) being the single-peaked likelihood of the sensory measurement for the relative 0° orientation, *p*(*m*_90_|θ) being the single-peaked likelihood of the sensory measurement for the relative 90° orientation, and *w*_AR_ being equal to $AR^{c\_stim}$. The size of the AR effect on the relative height of the 0° and 90° peaks is thus determined by a constant (i.e., *c*_stim_), representing the observer’s sensitivity to AR.

Each sensory measurement *m* is modeled as

(4) $$m=F\left(\theta +\delta_{stim}\right)+\delta_{sens},$$

with δ_*stim*_ representing the stimulus noise (added in stimulus space), δ_*sens*_ the sensory noise (added in sensory space), θ being the absolute orientation of the stimulus, and the transformation *F* being the cumulative distribution of the prior *p*(θ), which determines the stimulus-to-sensory mapping (Wei & Stocker, 2015). For each stimulus orientation θ_*i*_, *p*(*m*|θ_*i*_) can be computed according to (4) and the specific noise distributions. Both single-peaked likelihood functions, i.e., *p*(*m*_0_|θ) for *m*_0_ and *p*(*m*_90_|θ) for *m*_90_, are generated with the same level of stimulus noise (inversely represented in the model as stimulus precision: κ_stimL1_) and the same level of sensory noise (included in the model as sensory precision: κ_sensL1_). As described earlier in this article, the external stimulus noise (symmetric in stimulus space) is assumed to follow a von Mises (i.e., circular normal) distribution on the 180° (i.e., half-circular) orientation space with its mean at the actual stimulus orientation value in question and its precision being equal to κ_stimL1_. The internal sensory noise (symmetric in sensory space) is expected to follow a von Mises (i.e., circular normal) distribution on the 180° (i.e., half-circular) orientation space with its mean at the expected sensory measurement for the actual stimulus orientation value in question (based on the stimulus-to-sensory mapping, derived from the cumulative density function for the prior distribution) and its precision being equal to κ_*sensL*1_. For implementational details on the computation of the likelihood, we refer the interested reader to the model code, which is publicly available on OSF.

#### Posterior distribution and percept for the first lattice

To arrive at the posterior probability distribution for the perceived orientation of the first lattice (cf. Figure 3c), prior and likelihood are combined:

(5) $$p\left(\theta |m_{L1}\right)\propto p\left(\theta \right)\cdot p\left(m_{L1}|\theta \right).$$

From this posterior distribution, the probability of perceiving the relative 0° or 90° orientation can directly be deduced, for example, for the relative 0° orientation:

(6) $$\;p\left(\hat{\theta_{L1}}=0^{\circ }\right)=\frac{p(\theta =0^{\circ }|m_{L1})}{p\left(\theta =0^{\circ }|m_{L1}\right)+p\left(\theta =90^{\circ }|m_{L1}\right)}.$$

In case one wants to derive perceptual responses, one of the two dominant orientations can be sampled with the relative posterior probability at these orientations.

#### Perception of the second lattice

We model how an observer comes to perceive either the relative 0° , the relative 60° or the relative 120° orientation in the second lattice. Over trials, both the AR of the preceding first lattice and the absolute orientation of the lattices (same for first and second lattice) are varied. The distribution of absolute lattice orientations in the experiment is uniform, but the long-term natural stimulus distribution of orientations has peaks at both cardinal orientations. As in earlier Bayesian encoding models, we assume encoding resources to be allocated according to the stimulus distribution (uniform in the experiment or with peaks at the cardinals in the long term) so that stimulus values that occur more frequently are more accurately represented. Two types of noise are assumed: external stimulus noise and internal sensory noise. The external stimulus noise (symmetric in stimulus space) is assumed to follow a von Mises (i.e., circular normal) distribution on the 180° (i.e., half-circular) orientation space with its mean at the actual stimulus orientation value in question and its precision being equal to κ_stimL1_. The internal sensory noise (symmetric in sensory space) is expected to follow a von Mises (i.e., circular normal) distribution on the 180° (i.e., half-circular) orientation space with its mean at the expected sensory measurement for the actual stimulus orientation value in question (based on the stimulus-to-sensory mapping, derived from the cumulative density function for the prior distribution) and its precision being equal to κ_sensL1_. The described stimulus and sensory noise are jointly reflected in the noise of the observer’s representation of the stimulus orientation. The observer’s representation of the stimulus orientation (subject to the stimulus noise and sensory noise described above) is expected to be multimodal, with peaks at the relative 0° , relative 60° and relative 120° orientation. This multimodal distribution (with equal weighting for each of the three distributions) represents the likelihood, and is combined with a prior distribution to compute the posterior distribution for the second lattice. The prior distribution for the second lattice is a perceptual prior distribution, defined as a weighted combination of the prior distribution for the first lattice, indicating the long-term frequency of occurrence (i.e., uniform distribution or with peaks at the cardinals), and the recent perceptual history (i.e., a von Mises distribution with a precision of κ_percL1_ and its mean at the perceived orientation for the first lattice). From the posterior distribution, either a relative 0°, relative 60°, or relative 120° percept can be sampled with the probabilities depending on the relative probability of perceiving each of the three orientations.

#### Prior distribution for the second lattice

In the current version of the model, we assume two different frequency distributions: a stimulus frequency distribution affecting the stimulus-to-sensory mapping and a perceptual frequency distribution used in combination with the likelihood to form the posterior distribution. A version of the model that used the stimulus frequency distribution for both the stimulus-to-sensory mapping and the prior distribution could explain the occurrence of adaptation, but not the occurrence of the hysteresis effect.

##### The stimulus frequency distribution determines the stimulus-to-sensory mapping for the second lattice

The stimulus frequency distribution for the second lattice (cf. Figure 4a) is defined as a weighted mixture between the posterior for the first lattice (representing short-term context influences based on the stimulus evidence present) and the prior for the first lattice (representing longer-term context influences of the natural stimulus distribution; both a prior peaking at the cardinals and a uniform prior are tested, cf. above). If the weight of the posterior compared to that of the prior is increased (i.e., higher *w*_stimL1_), the stimulus prior will update more heavily based on the immediate stimulus history.

(7) $$\;p\left(\theta_{L2stim}\right)\propto p\left(\theta_{L1}\right)\cdot \left(1-w_{stimL1}\right)+p\left(\theta |m_{L1}\right)\cdot w_{stimL1}\;\;\;\;$$

##### The perceptual frequency distribution determines the prior for the second lattice

The perceptual frequency distribution for the second lattice (cf. Figure 4b) is defined as a weighted mixture between the prior for the first lattice (representing longer-term perceptual context; both a prior peaking at the cardinals and a uniform prior are tested, as discussed earlier in this article) and a single-peaked von Mises distribution around the perceived orientation of the first lattice. If the weight of the single-peaked von Mises distribution compared to that of the long-term perceptual frequency distribution is increased (i.e., higher *w*_percL1_), the perceptual prior will update more heavily based on the immediate perceptual history. Different from the stimulus frequency distribution, the perceptual prior thus includes direct information about the percept/decision/response concerning the first lattice. We assume the precision of the single-peaked von Mises distribution part of the perceptual prior (i.e., κ_percL1_) to be smaller than the stimulus or sensory precision for the second lattice (given that the percept for the first lattice is not visually present, this creates the possibility for more noise than for the second lattice, which is visually present).

(8) $$\begin{array}{ccc} & & \;p\left(\theta_{L2perc}\right)\propto p\left(\theta_{L1}\right)\cdot \left(1-w_{percL1}\right)+semicircular\;von\\ & & \;Mises\left(\hat{\theta_{L1}},\kappa_{percL1}\right)\cdot w_{percL1} \end{array}$$

#### Likelihood distribution for the second lattice

Given that the second dot lattice is hexagonal, it has three equally dominant orientations. Therefore, we assume the observer’s representation of the second lattice to be a combination of the sensory measurements for the 0°, 60°, and 120° orientations, with equal weight for all three sensory measurements. To arrive at a triple-peaked likelihood (cf. Figure 4c), we combine the likelihoods for all three sensory measurements (i.e., the sensory measurements for the relative 0°, 60°, and 120° orientation) with equal weights:

(9) $$\begin{array}{c} \;p\left(m_{L2}|\theta \right)\propto p\left(m_{0}|\theta \right)\cdot \frac{1}{3}+p\left(m_{60}|\theta \right)\cdot \frac{1}{3}+p\left(m_{120}|\theta \right)\cdot \frac{1}{3},\;\;\;\;\;\; \end{array}$$

with *p*(*m*_0_|θ), *p*(*m*_60_|θ), and *p*(*m*_120_|θ) being the single-peaked likelihoods of the sensory measurements for the relative 0°, 60°, and 120° orientation, respectively. As for the first lattice, each sensory measurement *m* is modeled as in (4), using the cumulative distribution of the stimulus prior (7) to determine the stimulus-to-sensory mapping. For each stimulus orientation θ_*i*_, *p*(*m*|θ_*i*_) can be computed according to (4) and the specific noise distributions. Each single-peaked likelihood function is generated with the same level of stimulus noise (inversely represented in the model as stimulus precision: κ_stimL2_) and the same level of sensory noise (included in the model as sensory precision: κ_sensL2_). As described earlier in this article, the external stimulus noise (symmetric in stimulus space) is assumed to follow a von Mises (i.e., circular normal) distribution on the 180° (i.e., half-circular) orientation space with its mean at the actual stimulus orientation value in question and its precision being equal to κ_stimL2_. The internal sensory noise (symmetric in sensory space) is expected to follow a von Mises (i.e., circular normal) distribution on the 180° (i.e., half-circular) orientation space with its mean at the expected sensory measurement for the actual stimulus orientation value in question (based on the stimulus-to-sensory mapping, derived from the cumulative density function for the prior distribution) and its precision being equal to κ_sensL2_. Given that the second lattice was presented more briefly than the first lattice (300 ms vs. 800 ms), we assume the sensory precision for the second lattice to be lower than the precision for the first lattice. For implementational details on the computation of the likelihood, we refer the reader to the model code, which is publicly available on OSF.

#### Posterior distribution and percept for the second lattice

To arrive at the posterior probability distribution for the perceived orientation of the second lattice (cf. Figure 4d), the *perceptual* prior distribution and the likelihood distribution are combined:

(10) $$p\left(\theta |m_{L2}\right)\propto p\left(\theta_{L2perc}\right)\cdot p\left(m_{L2}|\theta \right).$$

From this posterior distribution, the probability of perceiving the relative 0°, 60°, or 120° orientations can directly be deduced, for example, for the relative 0° orientation:

(11) $$\;p\left(\hat{\theta_{L2}}=0^{\circ }\right)=\frac{p(\theta =0^{\circ }|m_{L2})}{\begin{array}{c} p\left(\theta =0^{\circ }|m_{L2}\right)+p\left(\theta =60^{\circ }|m_{L2}\right)\\ \;+\;p(\theta =120^{\circ }|m_{L2}) \end{array}}.$$

In case one wants to derive perceptual responses, one of the three dominant orientations can be sampled with the relative posterior probability at these orientations.

### Free parameters in efficient Bayesian observer model

*c*_stim_ influences the strength of the effect of AR on the relative difference in height between the 0° and 90° peaks in the likelihood distribution for the first lattice. When *c*_stim_ is increased, AR more heavily influences the difference in height for the 0° and the 90° peak in the likelihood distribution for the first lattice.

κ_stimL1_ (i.e., stimulus precision for the first rectangular lattice) and κ_stimL2_ (i.e., stimulus precision for the second hexagonal lattice) influence the general precision of the likelihood peaks for the first and the second lattice, respectively. Stimulus precision does not alter the asymmetry of the likelihood distributions in stimulus space. When κ_*stim*_ is decreased, lower stimulus precision or, in other words, more external stimulus noise, is present.

κ_sensL1_ (i.e., sensory precision for the first lattice) and κ_sensL2_ (i.e., sensory precision for the second lattice) influence the asymmetry of the likelihood distributions for the first and the second lattice (in stimulus space), respectively. When κ_sens_ is decreased, lower sensory precision or thus more internal sensory noise is present. Given the difference in presentation time (i.e., 800 ms for the first and 300 ms for the second lattice), we assume κ_sensL1_ to be higher than κ_sensL2_.

*w*_stimL1_ (i.e., the weight of the posterior of the first lattice on the stimulus prior for the second lattice) determines the relative influence of the short-term effect of the first lattice on the stimulus prior for the second lattice compared to the influence of the long-term natural stimulus distribution.

*w*_percL1_ (i.e., the weight of the percept of the first lattice on the perceptual prior for the second lattice) determines the relative influence of the percept of the first lattice on the perceptual prior for the second lattice compared to a uniform distribution.

κ_percL1_ (i.e., the precision of the peak for the percept of the first lattice) reflects the precision of the von Mises distribution used in determining the perceptual prior for the second lattice.

### Model calculations and analyses

To investigate the effect of different model choices and parameters, we calculated the probabilities of perceiving the relative 0° orientation in the first and the second lattice for different versions of the general model described above. For each version of the model that we investigated, we calculated the probabilities for each possible trial, with the trial defined by a combination of the AR of the first lattice (i.e., 1.3^−1^, 1.2^−1^, 1.1^−1^, 1.0, 1.1, 1.2, and 1.3), and the percept of the first lattice (i.e., relative 0° or relative 90° orientation). When using a non-uniform natural stimulus distribution in the prior for the first lattice, we also calculated the probabilities for each absolute lattice orientation (i.e., from 1° to 180° in steps of 1°).^3^

Our first aim was to find a model and parameter combination that matched well with the average behavioral results found in Van Geert et al. (2022). Once this model version and parameter values was found, we manipulated each of the model parameters separately to investigate their effect on the expected probabilities of perceiving the relative 0° orientation in the first and the second lattice.

Our second aim was to introduce variation in some of the parameter values, to approximate the interindividual variation in effect size and direction found in the behavioral data for the dot lattices paradigm (Van Geert et al., 2022). We varied (a) the constant influencing the relation between the AR and differential height of the 0° and 90° peak in the likelihood for the first lattice (*c*_stim_), (b) the weight of the posterior of the first lattice on the stimulus prior for the second lattice (*w*_stimL1_), and (c) the weight of the percept of the first lattice on the perceptual prior for the second lattice (*w*_percL1_). To investigate whether we could reproduce the strong positive correlation between individuals’ hysteresis and adaptation effects found in Van Geert et al. (2022), we drew 75 individual parameter combinations for *c*_stim_, *w*_stimL1_, and *w*_percL1_ from a truncated multivariate normal distribution with means of 5, 6.5, and 5, a lower boundary of zero for all three parameters, an upper boundary of 10 for *w*_stimL1_ and *w*_percL1_, and the following variance-covariance matrix:

$$\begin{array}{c} \left[\begin{array}{ccc} 25 & 0 & 0\\ 0 & 9 & 8.95\\ 0 & 8.95 & 9 \end{array}\right].\; \end{array}$$

The *w*_stimL1_ and *w*_percL1_ parameters were then rescaled with a maximum of one instead of ten to match the zero-to-one range. We then calculated the probabilities of perceiving the relative 0° orientation in the first and the second lattice for all 75 parameter combinations and calculated the expected frequencies of each response given those probabilities.

To compare the variation in hysteresis and adaptation effects in the models to the variation in the behavioral results from Van Geert et al. (2022), and also to compare the observed correlation between individual hysteresis and adaptation effects, we conducted similar Bayesian analyses as in Van Geert et al. (2022) to the simulated data. More specifically, we estimated individual hysteresis and adaptation effects using a Bayesian multilevel binomial regression model predicting the percept of the second lattice, with the AR of the first lattice (*AR*) and the percept of the first lattice (*R*10) as fixed and random effects. To estimate the direct proximity effect, we used a Bayesian multilevel binomial regression model predicting the percept of the first lattice, with the AR of the first lattice (*AR*) as fixed and random effect. For more details on these Bayesian analyses, please consult the Supplementary Material as well as Van Geert et al. (2022).

## Results

### Approximation of average attractive and repulsive temporal context effects

After exploration of several parameter combinations, we were able to approximate the average behavioral results of Van Geert et al. (2022) with both a uniform (cf. Figure 5a) or a natural stimulus distribution prior for the first lattice (cf. Supplementary Figure 3) and the following parameter values: *c*_stim_ = 5, κ_stimL1_ = 20, κ_sensL1_ = 20, κ_stimL2_ = 20, κ_sensL2_ = 18, κ_percL1_ = 10, *w*_stimL1_ = 0.60, and *w*_percL1_ = 0.50. Whether a uniform prior distribution or a natural stimulus distribution was used as prior for the first lattice did not visibly influence the results. Given the considerable number of parameters, other parameter combinations could give results similar to the one proposed here. Therefore, we provide an online Shiny application in which the user can play with the different parameter values to test their effects, both on the trial and on the experiment level (https://elinevg.shinyapps.io/dotlatticesimulations/).

A version of the model using the stimulus prior in combination with the likelihood for the second lattice instead of the perceptual prior was able to predict a repulsive context effect of the previous stimulus evidence, but not the attractive effect of the previous percept (cf. Figure 5b). Although the predicted repulsive effect is only weak when using the same weights as in Figure 5a (cf. Supplementary Figure 4), this is a consequence of the parameter settings: as in this version of the model, the same distribution is used in the prior (resulting in prior attraction) and in the likelihood (resulting in likelihood repulsion), and *w*_stimL1_ and *w*_percL1_ are almost equal, attractive and repulsive effects largely cancel each other out. If *w*_stimL1_ is increased and *w*_percL1_ is decreased, a stronger repulsive effect is visible (cf. Figure 5b).

Is efficient encoding necessary to reproduce the behavioral results? A version of the model without efficient encoding was able to predict an attractive context effect of the previous percept^4^ (as the perceptual prior was still combined with the likelihood for the second lattice), but not the repulsive effect of the previous stimulus evidence, as that effect depends on the impact of the first lattice on the stimulus-to-sensory mapping and the likelihood of the second lattice (cf. Figure 5c).

### Effects of free parameters on attractive and repulsive temporal context effects

Here we start from the final efficient Bayesian observer model with a uniform prior for the first lattice and the parameters specified above and explore the effect of each parameter separately on the expected probabilities of perceiving the relative 0° orientation in the first and the second lattice. Under these settings, *c*_stim_ is the only parameter influencing the size of the direct proximity effect (i.e., the effect of the AR on the percept of the first lattice; cf. Figure 6a). Through its influence on the likelihood for the first lattice, *c*_stim_ also indirectly influences the size of the repulsive context effect of the AR on the second lattice (cf. Figure 7a).

Because κ_stimL1_ only decreases overall precision of the likelihood distribution for the first lattice (which increases the influence of the prior on the posterior) and a uniform prior distribution is used, a change in κ_stimL1_ does not have an influence on the relative posterior probabilities for the 0° and 90° orientation in the first lattice. Therefore, κ_stimL1_ does not influence the size of the proximity effect in case a uniform prior is used for the first lattice (cf. Figure 6b). In the expected probabilities for the second lattice, a higher stimulus precision for the first lattice (i.e., κ_stimL1_) results in slightly lower probabilities of perceiving the relative 0° orientation in the second lattice, especially for lower ARs (i.e.., in favor of the relative 0° orientation). In other words, a higher κ_stimL1_ thus results in a slightly stronger repulsive effect of the previous stimulus evidence (cf. Figure 7b).

Given a uniform prior distribution, also κ_sensL1_ does not influence the relative posterior probabilities for the 0° and 90° orientation in the first lattice. In other words, κ_sensL1_ does not influence the size of the proximity effect in case a uniform prior is used for the first lattice (cf. Figure 6c). In the expected probabilities for the second lattice, a higher sensory precision for the first lattice (i.e., κ_stimL1_) results in a slightly stronger repulsive effect of the previous stimulus evidence (cf. Figure 7c).

As can be seen in Figure 7d, increasing κ_stimL2_ slightly increases the expected probabilities for perceiving the 0° orientation in the second lattice overall, but more so for lower ARs. Hence, a higher κ_stimL2_ results in a slightly shallower adaptation effect (i.e., repulsive effect of the previous stimulus evidence). Increasing κ_sensL2_ leads to the opposite effect (cf. Figure 7e): the higher the sensory precision for the second lattice, the stronger the adaptation effect.

The more precise the peak in the perceptual prior for the second lattice, the higher the overall probability of perceiving the relative 0° orientation in the second lattice. Although the effect of κ_percL1_ is present regardless of the percept for the first lattice being the relative 0° or the relative 90° orientation, the effect of κ_percL1_ is larger for conditions in which the relative 0° orientation was perceived in the first lattice (cf. Figure 7f).

Increasing the weight of the previous stimulus evidence compared to the long-term uniform stimulus distribution (i.e., *w*_stimL1_) increases the size of the adaptation effect (cf. Figure 7g). Increasing the weight of the previous percept compared to the long-term uniform perceptual history (i.e., *w*_percL1_) increases the size of the hysteresis effect (cf. Figure 7h).

The effects of different parameter combinations can be further explored in the online Shiny application that we provide (https://elinevg.shinyapps.io/dotlatticesimulations/). In this application, the user can play with the different parameter values and combinations to test their effects, both on the trial and on the experiment level.

### Interindividual variation in proximity, hysteresis, and adaptation

When introducing interindividual variation in the parameter values for *c*_stim_, *w*_stimL1_, and *w*_percL1_, interindividual variation in proximity, hysteresis, and adaptation effects results. With the currently used parameter combinations, the size of the hysteresis and adaptation effects varied less in the simulation data than in the empirical data, but the simulated variation is plausible given the empirical data (cf. Figure 8 for average results and Figure 9 for individual simulation results). Furthermore, the same relation between hysteresis and adaptation effects is visible as in the empirical data: By generating *w*_stimL1_ and *w*_percL1_ in a positively correlated manner, we were able to reproduce the empirically found positive correlation between individuals’ attractive and repulsive temporal context effects (cf. Figure 10b). Different from the empirical results in Van Geert et al. (2022), the adaptation effect showed a strong negative correlation with the direct proximity effect in the simulation results and the hysteresis effect showed no correlation with the direct proximity effect (cf. Supplementary Figure 7).

## Discussion and conclusions

We tested whether the observed attractive and repulsive temporal context effects could be explained by an efficient Bayesian observer model (Wei & Stocker, 2015), which has previously been successfully applied to many different study designs involving non-ambiguous stimulus perception (e.g., Fritsche et al., 2020; Langlois et al., 2021; Wei & Stocker, 2015). The efficient Bayesian observer model assumes variable encoding precision of orientations in line with their frequency of occurrence (i.e., efficient encoding) and takes the dissimilarity between stimulus space and sensory space into account, which leads to asymmetric likelihood distributions as a result of uncertainty induced by internal sensory noise, and consequently the possibility for “anti-Bayesian” percepts biased away from the observer’s prior beliefs.

A hierarchical efficient Bayesian observer model including both a stimulus and a perceptual level was needed to explain the co-occurrence of both attractive and repulsive temporal context effects. The AR of the first lattice (i.e., the previous stimulus evidence) affected the percept of the second lattice via the stimulus-to-sensory mapping (i.e., efficient encoding) and the likelihood (i.e., likelihood repulsion) of the second lattice. The previous percept affected the perceptual prior for the second lattice and as a consequence the posterior probability of perceiving the relative 0° orientation in the second lattice (i.e., prior attraction). In other words, efficient encoding and likelihood repulsion on the stimulus level could explain the repulsive context effect, whereas perceptual prior attraction could explain the attractive temporal context effect when perceiving multistable dot lattices. This reasoning was confirmed based on simulations from model variants without efficient coding (showing only hysteresis) and without a perceptual prior (showing only adaptation). The conclusion that a hierarchical model including both a stimulus and a perceptual level is needed, is in line with Mao and Stocker (2024), who suggested the need to take higher-level representations into account to adequately model human subjects’ orientation percepts.

Not only the mean attractive and repulsive temporal context effects present in Van Geert et al. (2022) could be reproduced using a hierarchical efficient Bayesian observer model, also plausible variation in effect size and direction could be derived by varying (a) the constant influencing the relation between the AR and differential height of the 0° and 90° peak in the likelihood for the first lattice (*c*_stim_), (b) the weight of the posterior of the first lattice on the stimulus prior for the second lattice (*w*_stimL1_), and (c) the weight of the percept of the first lattice on the perceptual prior for the second lattice (*w*_percL1_). Furthermore, the hierarchical efficient Bayesian observer model could reproduce the empirically observed strong positive correlation between individuals’ attractive and repulsive effects (Van Geert et al., 2022), by assuming a positive correlation between temporal integration constants at the stimulus and the perceptual level. That is, individuals who weight the previous stimulus evidence more highly in relation to the long-term stimulus context will also weight the previous percept more highly in relation to the long-term perceptual context than individuals who weight the previous stimulus evidence less highly. Assuming separate but correlated temporal integration constants at the stimulus and the perceptual level is not implausible in our opinion. For instance, Fritsche et al. (2020) found better performance for a model with different integration time constants for prior and likelihood than for a model that used the same parameters for both. Different from the successful reproduction of the high positive correlation between attractive and repulsive temporal context effects, the correlations between the temporal context effects and the direct proximity effect did not match those observed in the empirical data. Follow-up research may aim to find parameter combinations that provide a closer match to those aspects of the empirical data.

Whereas earlier models induced a direct effect of the previous stimulus evidence on the likelihood distribution for the second lattice (Schwiedrzik et al., 2014), or posited a persistent bias for an absolute orientation but did not model the origin of the bias (Gepshtein & Kubovy, 2005), the current efficient Bayesian observer model provides a more complete process model of how previous percept and stimulus evidence can influence multistable dot lattice perception. We consider changes in the frequency of occurrence (in this case in the short term) and consequently in the prior distribution and the stimulus-to-sensory mapping a conceptually plausible explanation for the co-occurrence of separate but related short-term attractive and repulsive temporal context effects. The currently proposed model thus integrates explanations for both mechanisms in one coherent (hierarchical) theory, which was not the case in the earlier models of the dot lattice paradigm (Gepshtein & Kubovy, 2005; Schwiedrzik et al., 2014). Assuming separate but related processes underlying both context effects present at multiple hierarchical levels (i.e., likelihood repulsion on the stimulus level and prior attraction on the perceptual level, related through their dependence on the posterior for the first lattice), provides an intermediate position, in between researchers positing one single mechanism underlying both effects and researchers confirming differences in the characteristics of both effects. Furthermore, the idea of separate but related processes inherent in the efficient Bayesian observer model is highly compatible with the empirically observed high correlation between individuals’ attractive and repulsive temporal context effects as observed in Van Geert et al. (2022).

It is highly likely that our visual system takes more previous stimulus evidence or percepts into account than only one stimulus back. Another possible follow-up is, therefore, to take changes throughout the entire experiment into account when modeling the behavioral data resulting from the multistable dot lattice paradigm. However, the presence of a mask in between trials makes modeling this process conceptually more complex. Furthermore, when modeling the results for the current paradigm, going only one trial back, was enough to replicate the behavioral effects. It was thus not necessary to go more than one trial back to successfully account for the co-occurrence of both effects.

The current computational model can serve to inspire new experimentation. The model can generate quantitative predictions that can be tested in new experiments: for example, sensory noise can be manipulated using exposure time or stimulus contrast, the alignment of the dots in the lattice can be decreased to lower stimulus precision, or a longer inter-stimulus interval could be introduced to weaken the influence of the first lattice. Also, the same modeling approach can be adapted to other tasks measuring temporal context effects with different multistable stimuli.

To conclude, a hierarchical efficient Bayesian observer model including both a stimulus and a perceptual level can explain repulsive temporal context effects in multistable dot lattice perception via efficient encoding and likelihood repulsion, and attractive effects via perceptual prior attraction. This conclusion is in line with the conclusion of Mao and Stocker (2024), who suggested the need for considering the complex hierarchical structure of the brain, by also taking the higher-level representation into account to adequately model human subjects’ response behavior.

## Supplementary Material

Supplement 1

## Footnotes

1 For an overview of all R packages used, see the Supplementary Material.

2 In most figures, we show the results based on internal sensory noise distributions with their means at the expected sensory measurements (i.e., the relative 0° and relative 90° orientations). However, we also simulated a model with sensory measurements sampled from the probability distribution of sensory measurements given the actual stimulus orientation (cf. Supplementary Figure 5).

3 When using a uniform prior distribution for the first lattice, the absolute lattice orientation does not influence the posterior probabilities for the first or the second lattice.

4 When the participant perceived the 90° orientation in the first lattice, no attractive context effect can be present as the 90° orientation of the first lattice is never close to one of the three orientations present in the second lattice (0°, 60°, and 120°). Therefore, hysteresis, i.e., an attractive context effect or having the same percept in both lattices, can only occur when the previous percept was the 0° orientation and the probability of perceiving the 0° orientation in the second lattice is at chance level when the previous percept was the 90° orientation.
