# Supplementary material for: An efficient Bayesian observer model of attractive and repulsive temporal context effects when perceiving multistable dot lattices
Source: J Vis. 2024 Apr 18;24(4):18. doi: 10.1167/jov.24.4.18 (PMC11037491; doi:10.1167/jov.24.4.18)
Supplement: Supplement 1 [file jovi-24-4-18_s001.pdf]

# Supplemental Appendix for An efficient Bayesian observer model of attractive and repulsive temporal context effects when perceiving multistable dot lattices

Eline Van Geert[†,1], Tina Ivančir[†,1], & Johan Wagemans[1]

[1] KU Leuven, Belgium

## Author Note

The authors made the following contributions. Eline Van Geert: Conceptualization, Data curation, Formal analysis, Funding acquisition, Investigation, Methodology, Project administration, Resources, Software, Validation, Visualization, Writing - original draft, Writing - review & editing; Tina Ivančir: Conceptualization, Formal analysis, Investigation, Methodology, Software, Visualization, Writing - review & editing; Johan Wagemans: Conceptualization, Funding acquisition, Supervision, Writing - review & editing. [†] Eline Van Geert and Tina Ivančir contributed equally to this work.

Correspondence concerning this article should be addressed to Eline Van Geert, Laboratory of Experimental Psychology, KU Leuven, Tiensestraat 102 - box 3711, 3000 Leuven. Telephone: +32 16 37 34 80. E-mail: eline.vangeert@kuleuven.be

# Appendix

## Supplementary Figures and Information

As indicated in the Methods section, for all our analyses we used R (Version 4.3.1; R Core Team, 2021) and the R-packages *BayesFactor* (Morey & Rouder, 2018), *brms* (Version 2.19.0; Bürkner, 2017, 2018, 2021), *coda* (Version 0.19.4; Plummer, Best, Cowles, & Vines, 2006), *cowplot* (Version 1.1.1; Wilke, 2020), *devtools* (Version 2.4.5; Wickham, Hester, Chang, & Bryan, 2021), *doParallel* (Version 1.0.17; Corporation & Weston, 2020), *dplyr* (Version 1.1.2; Wickham, François, Henry, & Müller, 2021), *ellipse* (Version 0.5.0; Murdoch & Chow, 2020), *forcats* (Version 1.0.0; Wickham, 2021a), *foreach* (Version 1.5.2; Microsoft & Weston, 2020), *GGally* (Version 2.1.2; Schloerke et al., 2021), *ggdist* (Version 3.3.0; Kay, 2021a), *ggforce* (Pedersen, 2021), *ggnewscale* (Version 0.4.9; Campitelli, 2022), *ggplot2* (Version 3.4.2; Wickham, 2016), *ggstance* (Version 0.3.6; Henry, Wickham, & Chang, 2020), *glue* (Version 1.6.2; Hester & Bryan, 2021), *gmm* (Version 1.8; Chaussé, 2010), *here* (Version 1.0.1; Müller, 2020), *iterators* (Version 1.0.14; Analytics & Weston, 2020), *kableExtra* (Version 1.3.4; Zhu, 2021), *knitr* (Version 1.43; Xie, 2015), *lubridate* (Version 1.9.2; Grolemund & Wickham, 2011), *MASS* (Version 7.3.60; Venables & Ripley, 2002), *Matrix* (Version 1.5.4.1; Bates & Maechler, 2021), *mvtnorm* (Version 1.2.3; Genz & Bretz, 2009; Wilhelm & G, 2022), *papaja* (Version 0.1.1; Aust & Barth, 2020), *patchwork* (Version 1.1.3; Pedersen, 2020), *purrr* (Version 1.0.1; Henry & Wickham, 2020), *Rcpp* (Eddelbuettel & Balamuta, 2018; Version 1.0.11; Eddelbuettel & François, 2011), *readr* (Version 2.1.4; Wickham, Hester, & Bryan, 2021), *rstan* (Version 2.26.22; Stan Development Team, 2021), *sandwich* (Zeileis, 2004, 2006; Version 3.0.2; Zeileis, Köll, & Graham, 2020), *StanHeaders* (Version 2.26.27; Stan Development Team, 2020), *stringr* (Version 1.5.0; Wickham, 2019), *tibble* (Version 3.2.1; Müller & Wickham, 2021), *tidybayes* (Version 3.0.4; Kay, 2021b), *tidyr* (Version 1.3.0; Wickham, 2021b), *tidyverse* (Version 2.0.0; Wickham et al., 2019), *tinylabels* (Version 0.2.3; Barth, 2022), *tmvtnorm* (Version 1.5; Wilhelm & G, 2022), *truncnorm* (Mersmann, Trautmann, Steuer, & Bornkamp, 2018), and *usethis* (Version 2.2.2; Wickham, Bryan, & Barrett, 2021).

## Visualization of prior, likelihood, and posterior distributions for one trial

Figure A1 visualizes the prior, likelihood, and posterior distributions for the first lattice in a single trial of the dot lattices paradigm, given a model with a prior stimulus distribution incorporating the more frequent occurrence of cardinal compared to oblique orientations. Figure A2 visualizes the prior, likelihood, and posterior distributions for the second lattice in a single trial of the dot lattices paradigm, given the same natural stimulus distribution for the prior of the first lattice.

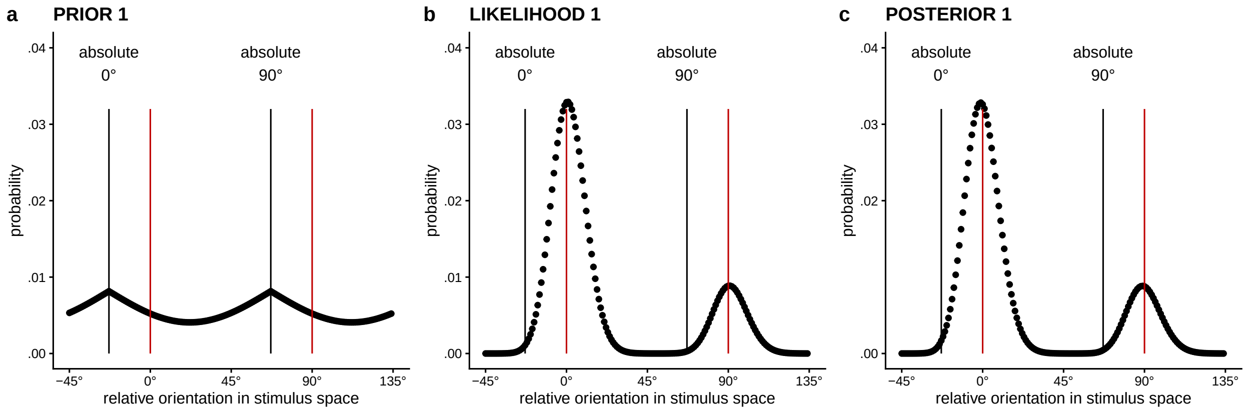


*Figure A1.* (a) Natural stimulus prior for the first lattice, defined by the formula: $p(\theta) = c_0(2 - |sin(2\theta)|)$, in which $\theta$ represents the orientation value and $c_0$ the normalizing constant. (b) Example of likelihood 1 defined in the stimulus space, for a first lattice with an absolute lattice orientation of 23° and AR = 1.3ˆ-1, which favors the relative 0° orientation. Consequently, the two likelihood peaks are skewed away from the absolute cardinal orientations indicated with the black vertical lines. (c) Posterior distribution for the first lattice. Based on the difference in height of the peaks for the relative 0° and 90° orientation, i.e., p(0°) and p(90°), the probability of a 0° or 90° response can be determined. *Note.* The red vertical lines in the graph are placed at the two dominant relative 0° and 90° orientations in the lattice. The black vertical lines label the absolute 0° and 90° orientations.

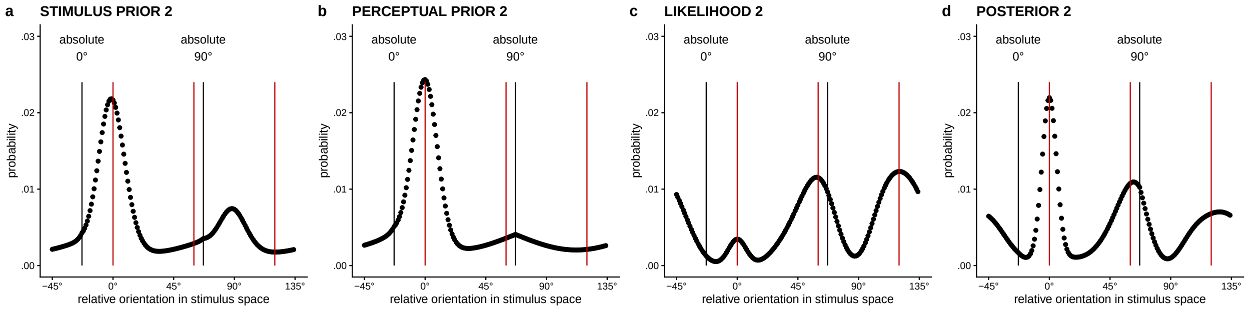


*Figure A2.* (a) Stimulus prior for the second lattice given a first lattice with an absolute lattice orientation of 23° and with AR = 1.3ˆ-1, which favors the relative 0° orientation. (b) Perceptual prior for the second lattice, given the relative 0° orientation was perceived in the first lattice. (c) Likelihood distribution defined in the stimulus space for the second lattice. This distribution is influenced by the stimulus prior for the second lattice (and hence the aspect ratio of the first lattice) via the stimulus-to-sensory mapping. (d) Posterior distribution for the second lattice, combining perceptual prior and likelihood for the second lattice. Based on the difference in height of the peaks for the relative 0°, 60°, and 120° orientation, the probability of a 0°, 60°, or 120° response can be determined. *Note.* The red vertical lines in the graph are placed at the three dominant relative 0°, 60°, and 120° orientations in the lattice. The black vertical lines label the absolute 0° and 90° orientations.

**Supplementary Figures related to the Approximation of average attractive and repulsive temporal context effects**

Figure A3 visualizes the logit probability of perceiving the relative 0° orientation based on efficient Bayesian observer model with a natural stimulus distribution prior for the first lattice. Figure A4 visualizes the logit probability of perceiving the relative 0° orientation based on efficient Bayesian observer model without a perceptual prior and with the same parameters as in Figure 5a. Figure A5 visualizes the logit probability of perceiving the relative 0° orientation in the first lattice and the second lattice, based on (a) an efficient Bayesian observer model with a flat prior distribution for the first lattice and the following parameters: $c_{\text{stim}} = 5$, $\kappa_{\text{stimL1}} = 20$, $\kappa_{\text{sensL1}} = 20$, $\kappa_{\text{stimL2}} = 20$, $\kappa_{\text{sensL2}} = 18$, $\kappa_{\text{percL1}} = 10$, $w_{\text{stimL1}} = 0.60$, and $w_{\text{percL1}} = 0.50$, (b) the same model as in (a) but sampling percepts for the first and the second lattice rather than directly using the posterior probability, and (c) the same model as in (a) but sampling the sensory measurements rather than assuming the expected sensory measurements for the first and the second lattice and sampling percepts rather than using the posterior probability.

**Supplementary information concerning Bayesian analyses of interindividual variation in simulation data**

We estimated individual hysteresis and adaptation effects using a Bayesian multilevel binomial regression model predicting the percept of the second lattice, with aspect ratio of the first lattice ($AR$) and the percept of the first lattice ($R10$) as fixed and random effects. To estimate the direct proximity effect (i.e., the direct effect of aspect ratio on perception of the first lattice), we used a Bayesian multilevel binomial regression model predicting the percept of the first lattice, with aspect ratio of the first lattice ($AR$) as fixed and random effect.

The model for the first lattice included fixed and individual random effects for aspect ratio $AR$ (i.e., proximity effect), and individual random intercepts. This model can be formulated as follows:

$$freq(r1 = 0^{\circ})|trials(n) \sim Intercept + AR + (Intercept + AR \mid participant).$$

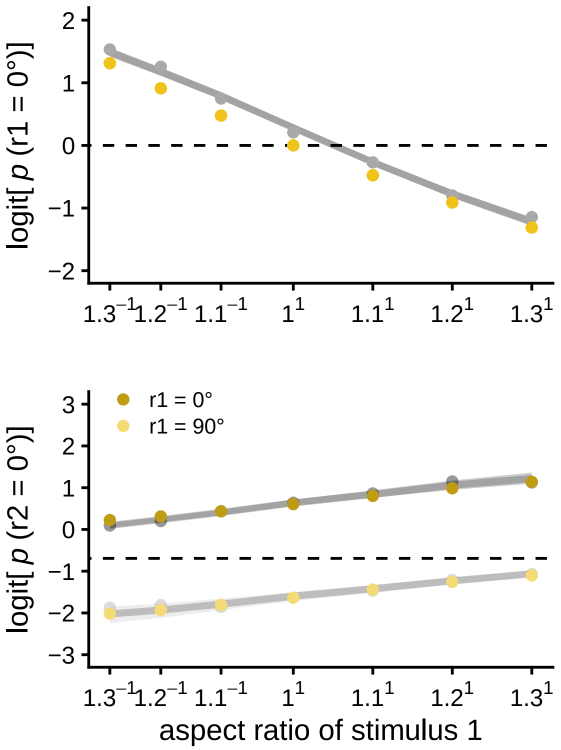


*Figure A3.* Visualization of the logit probability to perceive the relative 0° orientation in (a) the first lattice and (b) the second lattice, based on an efficient Bayesian observer model with a natural stimulus distribution prior for the first lattice and the following parameters: $c_{\text{stim}} = 5$, $\kappa_{\text{stimL1}} = 20$, $\kappa_{\text{sensL1}} = 20$, $\kappa_{\text{stimL2}} = 20$, $\kappa_{\text{sensL2}} = 18$, $\kappa_{\text{percL1}} = 10$, $w$~stimL1 = 0.60, and $w_{\text{percL1}} = 0.50$. The yellow dots indicate the probabilities based on the model. The behavioral results and the estimated effects based on the behavioral results of Van Geert, Moors, Haaf, and Wagemans (2022), averaged across participants, are indicated in dark grey for r1 = 0° and light grey for r1 = 90°.

To determine the size of the proximity effect, we used the individual estimates for the effect of the aspect ratio of the first lattice on the percept of the first lattice.

The model for the second lattice thus included fixed and individual random effects for percept in the first lattice *R10* (i.e., hysteresis effect) as well as aspect ratio in the first lattice *AR* (i.e., adaptation effect), and individual random intercepts. This model can be formulated as follows:

$$freq(r2 = 0°)|trials(n) \sim Intercept + AR + R10 + (Intercept + AR + R10 \mid participant).$$

To determine the size of the hysteresis effect, we used the individual estimates for the effect of the percept of the first lattice on the percept of the second lattice. To determine the size of the adaptation effect, we used the individual estimates for the effect of aspect ratio of the first lattice

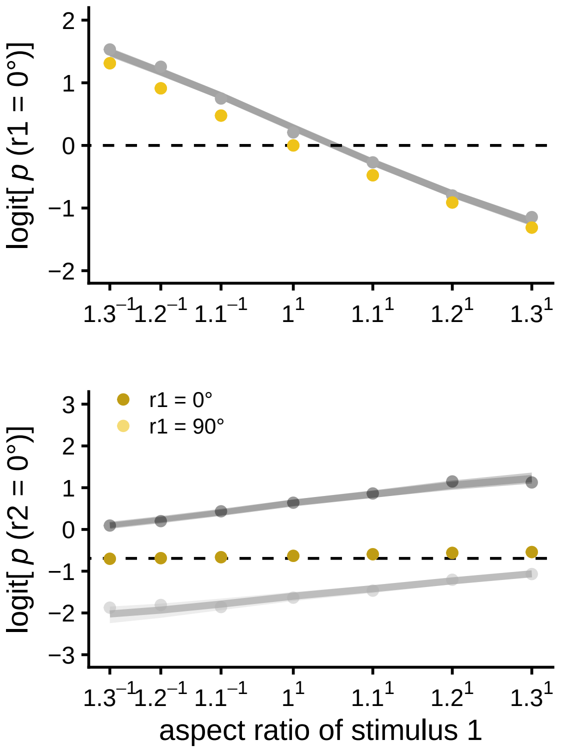


*Figure A4*. Visualization of the logit probability to perceive the relative 0° orientation in (a) the first lattice and (b) the second lattice, based on an efficient Bayesian observer model without a perceptual prior for the second lattice, a flat prior distribution for the first lattice, and the following parameters: $c_{\text{stim}} = 5$, $\kappa_{\text{stimL1}} = 20$, $\kappa_{\text{sensL1}} = 20$, $\kappa_{\text{stimL2}} = 20$, $\kappa_{\text{sensL2}} = 18$, $\kappa_{\text{percL1}} = 10$, $w_{\text{stimL1}} = 0.60$, and $w_{\text{percL1}} = 0.50$. The yellow dots indicate the probabilities based on the model. In the visualization for the second lattice, the dark and light yellow dots lay on top of each other. The behavioral results and the estimated effects based on the behavioral results of Van Geert et al. (2022), averaged across participants, are indicated in dark grey for r1 = 0° and light grey for r1 = 90°.

on the percept of the second lattice. To have an estimate of the strength of the correlation between the size of individuals' hysteresis and adaptation effects, we report the mean and 95% HDCI for the correlation between estimated individual hysteresis and adaptation effects, based on the full model described above.

In both the model for the first and for the second lattice, centered aspect ratio was used, which means that a value of zero corresponds to an aspect ratio of 1, a value of $1.1^{-1}$ -1 (i.e., ≈ -0.09) corresponds to $1.1^{-1}$, and a value of $1.1^{1}$ -1 (i.e., 0.10) to an aspect ratio of 1.1.

Figure A6 visualizes the priors we specified for the fixed effects, for the standard deviation of the random effects, and for the correlation matrix.

We fitted these models of perceived L1 and perceived L2 orientation using brms (Bürkner, 2017, 2018). We used 4 chains with 20000 iterations each with the default number of warmup

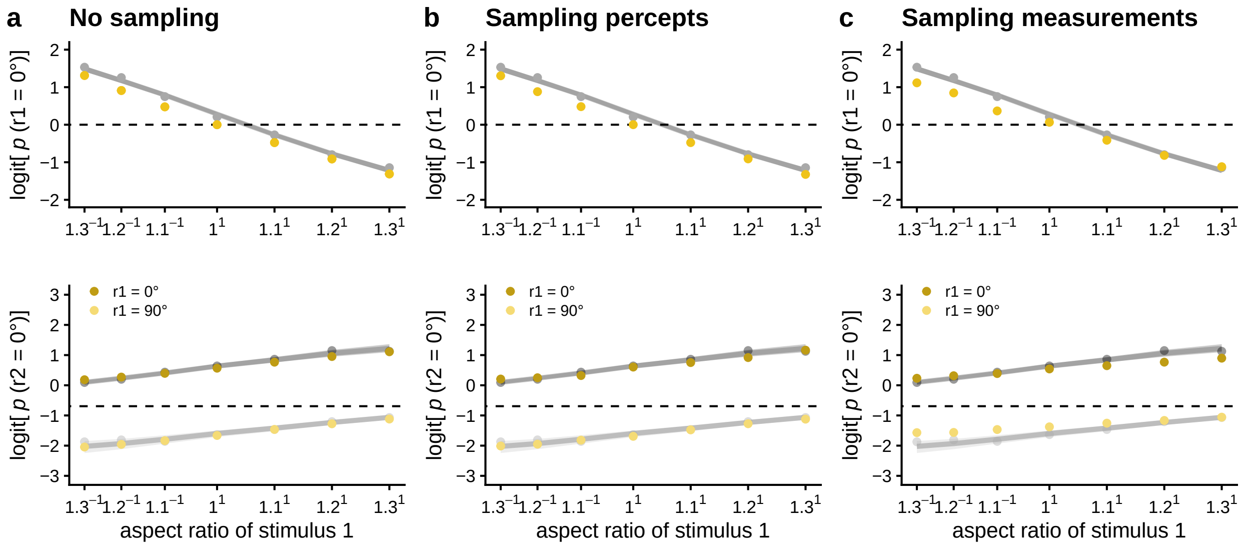


*Figure A5.* Visualization of the logit probability to perceive the relative 0° orientation in the first lattice and the second lattice, based on (a) an efficient Bayesian observer model with a flat prior distribution for the first lattice and the following parameters: $c_{\text{stim}} = 5$, $\kappa_{\text{stimL1}} = 20$, $\kappa_{\text{sensL1}} = 20$, $\kappa_{\text{stimL2}} = 20$, $\kappa_{\text{sensL2}} = 18$, $\kappa_{\text{percL1}} = 10$, $w_{\text{stimL1}} = 0.60$, and $w_{\text{percL1}} = 0.50$, (b) the same model as in (a) but sampling percepts for the first and the second lattice rather than directly using the posterior probability, and (c) the same model as in (a) but sampling the sensory measurements rather than assuming the expected sensory measurements for the first and the second lattice and sampling percepts rather than using the posterior probability. The yellow dots indicate the expected probabilities based on the model. The behavioral results and the estimated effects based on the behavioral results of Van Geert et al. (2022), averaged across participants, are indicated in dark grey for r1 = 0° and light grey for r1 = 90°. In (b) and (c), 6750 trials per aspect ratio or 47250 trials in total were included in the simulations, which equals the number of trials for 75 participants that each conduct 90 trials per aspect ratio or 630 trials in total, as was the case in the empirical study of Van Geert et al. (2022).

iterations per chain. For any other sampling specifications we used the default settings. For further details on these analyses and those for calculating the correlation between individuals' proximity, hysteresis, and adaptation estimates, please consult Van Geert et al. (2022).

**Supplementary Figure concerning interindividual variation in proximity, hysteresis, and adaptation**

Figure A7 visualizes the correlation of estimated individual hysteresis and adaptation effects concerning the second lattice with estimated individual proximity effects concerning the first lattice for the empirical data collected in Van Geert et al. (2022) and for the simulation results.

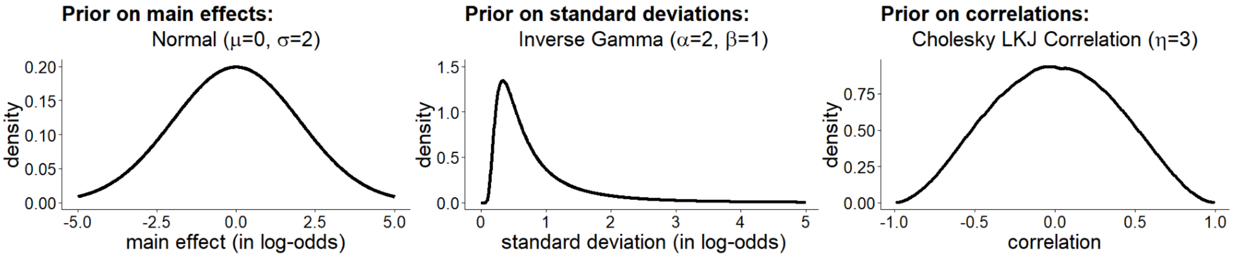


*Figure A6.* Illustration of priors used in the model predicting the percept of L1 and L2. Reprinted from Van Geert et al. (2022)

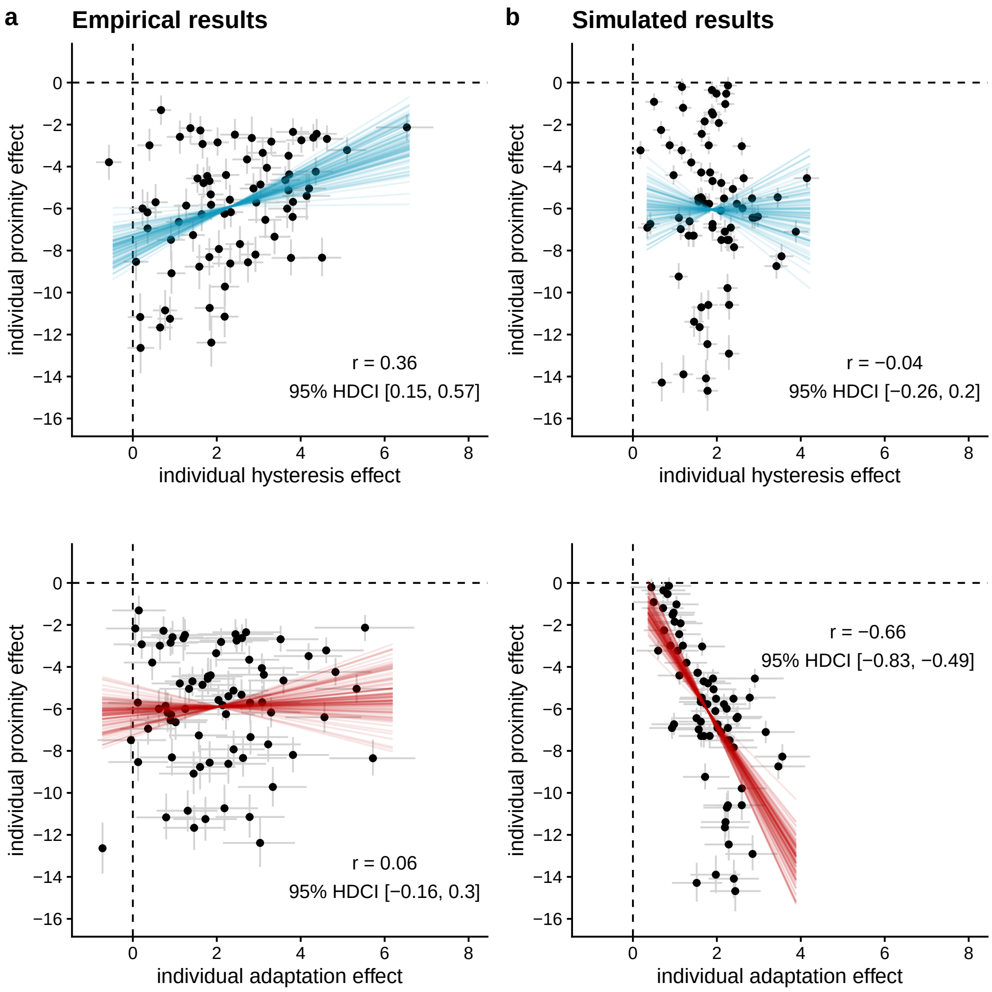


*Figure A7.* Correlation of estimated individual hysteresis and adaptation effects concerning the second lattice with estimated individual proximity effects concerning the first lattice for (a) the empirical data collected in Van Geert et al. (2022) and (b) the simulation results.

## References Appendix

Analytics, R., & Weston, S. (2020). *Iterators: Provides iterator construct*. Retrieved from https://CRAN.R-project.org/package=iterators

Aust, F., & Barth, M. (2020). *papaja: Create APA manuscripts with R Markdown*. Retrieved from https://github.com/crsh/papaja

Barth, M. (2022). *tinylabels: Lightweight variable labels*. Retrieved from https://cran.r-project.org/package=tinylabels

Bates, D., & Maechler, M. (2021). *Matrix: Sparse and dense matrix classes and methods*. Retrieved from https://CRAN.R-project.org/package=Matrix

Bürkner, P.-C. (2017). brms: An R package for Bayesian multilevel models using Stan. *Journal of Statistical Software*, *80*(1), 1–28. https://doi.org/10.18637/jss.v080.i01

Bürkner, P.-C. (2018). Advanced Bayesian multilevel modeling with the R package brms. *The R Journal*, *10*(1), 395–411.

Bürkner, P.-C. (2021). Bayesian item response modeling in R with brms and Stan. *Journal of Statistical Software*, *100*(5), 1–54. https://doi.org/10.18637/jss.v100.i05

Campitelli, E. (2022). *Ggnewscale: Multiple fill and colour scales in 'ggplot2'*. Retrieved from https://CRAN.R-project.org/package=ggnewscale

Chaussé, P. (2010). Computing generalized method of moments and generalized empirical likelihood with R. *Journal of Statistical Software*, *34*(11), 1–35. https://doi.org/10.18637/jss.v034.i11

Corporation, M., & Weston, S. (2020). *doParallel: Foreach parallel adaptor for the 'parallel' package*. Retrieved from https://CRAN.R-project.org/package=doParallel

Eddelbuettel, D., & Balamuta, J. J. (2018). Extending extitR with extitC++: A Brief Introduction to extitRcpp. *The American Statistician*, *72*(1), 28–36. https://doi.org/10.1080/00031305.2017.1375990

Eddelbuettel, D., & François, R. (2011). Rcpp: Seamless R and C++ integration. *Journal of Statistical Software*, *40*(8), 1–18. https://doi.org/10.18637/jss.v040.i08

Genz, A., & Bretz, F. (2009). *Computation of multivariate normal and t probabilities*. Heidelberg: Springer-Verlag.

Grolemund, G., & Wickham, H. (2011). Dates and times made easy with lubridate. *Journal of Statistical Software*, *40*(3), 1–25. Retrieved from https://www.jstatsoft.org/v40/i03/

Henry, L., & Wickham, H. (2020). *Purrr: Functional programming tools*. Retrieved from https://CRAN.R-project.org/package=purrr

Henry, L., Wickham, H., & Chang, W. (2020). *Ggstance: Horizontal 'ggplot2' components*. Retrieved from https://CRAN.R-project.org/package=ggstance

Hester, J., & Bryan, J. (2021). *Glue: Interpreted string literals*. Retrieved from https://CRAN.R-project.org/package=glue

Kay, M. (2021a). *ggdist: Visualizations of distributions and uncertainty*. https://doi.org/10.5281/zenodo.3879620

Kay, M. (2021b). *tidybayes: Tidy data and geoms for Bayesian models*. https://doi.org/10.5281/zenodo.1308151

Mersmann, O., Trautmann, H., Steuer, D., & Bornkamp, B. (2018). *Truncnorm: Truncated normal distribution*. Retrieved from https://CRAN.R-project.org/package=truncnorm

Microsoft, & Weston, S. (2020). *Foreach: Provides foreach looping construct*. Retrieved from https://CRAN.R-project.org/package=foreach

Morey, R. D., & Rouder, J. N. (2018). *BayesFactor: Computation of bayes factors for common designs*. Retrieved from https://CRAN.R-project.org/package=BayesFactor

Müller, K. (2020). *Here: A simpler way to find your files*. Retrieved from https://CRAN.R-project.org/package=here

Müller, K., & Wickham, H. (2021). *Tibble: Simple data frames*. Retrieved from https://CRAN.R-project.org/package=tibble

Murdoch, D., & Chow, E. D. (2020). *Ellipse: Functions for drawing ellipses and ellipse-like confidence regions*. Retrieved from https://CRAN.R-project.org/package=ellipse

Pedersen, T. L. (2020). *Patchwork: The composer of plots*. Retrieved from

https://CRAN.R-project.org/package=patchwork

Pedersen, T. L. (2021). *Ggforce: Accelerating 'ggplot2'*. Retrieved from https://CRAN.R-project.org/package=ggforce

Plummer, M., Best, N., Cowles, K., & Vines, K. (2006). CODA: Convergence diagnosis and output analysis for MCMC. *R News*, *6*(1), 7–11. Retrieved from https://journal.r-project.org/archive/

R Core Team. (2021). *R: A language and environment for statistical computing*. Vienna, Austria: R Foundation for Statistical Computing. Retrieved from https://www.R-project.org/

Schloerke, B., Cook, D., Larmarange, J., Briatte, F., Marbach, M., Thoen, E., … Crowley, J. (2021). *GGally: Extension to 'ggplot2'*. Retrieved from https://CRAN.R-project.org/package=GGally

Stan Development Team. (2020). *StanHeaders: Headers for the R interface to Stan*. Retrieved from https://mc-stan.org/

Stan Development Team. (2021). *RStan: The R interface to Stan*. Retrieved from https://mc-stan.org/

Van Geert, E., Moors, P., Haaf, J., & Wagemans, J. (2022). Same stimulus, same temporal context, different percept? Individual differences in hysteresis and adaptation when perceiving multistable dot lattices. *I-Perception*, *13*(4), 20416695221109300. https://doi.org/10.1177/20416695221109300

Venables, W. N., & Ripley, B. D. (2002). *Modern applied statistics with s* (Fourth). New York: Springer. Retrieved from http://www.stats.ox.ac.uk/pub/MASS4/

Wickham, H. (2016). *ggplot2: Elegant graphics for data analysis*. Springer-Verlag New York. Retrieved from https://ggplot2.tidyverse.org

Wickham, H. (2019). *Stringr: Simple, consistent wrappers for common string operations*. Retrieved from https://CRAN.R-project.org/package=stringr

Wickham, H. (2021a). *Forcats: Tools for working with categorical variables (factors)*. Retrieved from https://CRAN.R-project.org/package=forcats

Wickham, H. (2021b). *Tidyr: Tidy messy data*. Retrieved from https://CRAN.R-project.org/package=tidyr

Wickham, H., Averick, M., Bryan, J., Chang, W., McGowan, L. D., François, R., … Yutani, H. (2019). Welcome to the tidyverse. *Journal of Open Source Software*, *4*(43), 1686. https://doi.org/10.21105/joss.01686

Wickham, H., Bryan, J., & Barrett, M. (2021). *Usethis: Automate package and project setup*. Retrieved from https://CRAN.R-project.org/package=usethis

Wickham, H., François, R., Henry, L., & Müller, K. (2021). *Dplyr: A grammar of data manipulation*. Retrieved from https://CRAN.R-project.org/package=dplyr

Wickham, H., Hester, J., & Bryan, J. (2021). *Readr: Read rectangular text data*. Retrieved from https://CRAN.R-project.org/package=readr

Wickham, H., Hester, J., Chang, W., & Bryan, J. (2021). *Devtools: Tools to make developing r packages easier*. Retrieved from https://CRAN.R-project.org/package=devtools

Wilhelm, S., & G, M. B. (2022). *tmvtnorm: Truncated multivariate normal and student t distribution*. Retrieved from https://CRAN.R-project.org/package=tmvtnorm

Wilke, C. O. (2020). *Cowplot: Streamlined plot theme and plot annotations for 'ggplot2'*. Retrieved from https://CRAN.R-project.org/package=cowplot

Xie, Y. (2015). *Dynamic documents with R and knitr* (2nd ed.). Boca Raton, Florida: Chapman; Hall/CRC. Retrieved from https://yihui.org/knitr/

Zeileis, A. (2004). Econometric computing with HC and HAC covariance matrix estimators. *Journal of Statistical Software*, *11*(10), 1–17. https://doi.org/10.18637/jss.v011.i10

Zeileis, A. (2006). Object-oriented computation of sandwich estimators. *Journal of Statistical Software*, *16*(9), 1–16. https://doi.org/10.18637/jss.v016.i09

Zeileis, A., Köll, S., & Graham, N. (2020). Various versatile variances: An object-oriented implementation of clustered covariances in R. *Journal of Statistical Software*, *95*(1), 1–36. https://doi.org/10.18637/jss.v095.i01

Zhu, H. (2021). *kableExtra: Construct complex table with 'kable' and pipe syntax*. Retrieved from https://CRAN.R-project.org/package=kableExtra
